# *Weizmannia coagulans* BC99 regulates oxidative stress and serum metabolic pathways to improve allergic rhinitis: a randomized, double-blind, placebo-controlled trial

**DOI:** 10.3389/fimmu.2025.1654724

**Published:** 2025-10-24

**Authors:** Xuan Li, Zhidong Han, Kaihong Wu, Bingchai Lin, Saman Azeem, Yiru Jiang, Yao Dong, Zhonghui Gai, Shuguang Fang, Ying Wu, Shaobin Gu

**Affiliations:** ^1^ College of Food and Bioengineering, Henan University of Science and Technology, Luoyang, China; ^2^ Henan Engineering Research Center of Food Microbiology, Luoyang, China; ^3^ Guangdong Yi Chao Biological Technology Co., Ltd., Shantou, China; ^4^ Department of Research and Development, Wecare Probiotics Co., Ltd., Suzhou, China; ^5^ Henan Engineering Research Center of Food Material, Henan University of Science and Technology, Luoyang, China

**Keywords:** *Weizmannia coagulans* BC99, allergic rhinitis, oxidative stress, serum metabolism, immune factors

## Abstract

**Background:**

Probiotics have demonstrated potential application value in alleviating allergic diseases, but their mechanisms of action remain to be explored. This study aimed to investigate the clinical efficacy of probiotics in patients with allergic rhinitis (AR) and explore the underlying mechanisms.

**Methods:**

We evaluated the clinical intervention efficacy of *Weizmannia* coagulans BC99 (BC99) in adults with AR through a randomized, double-blind, placebo-controlled trial. Seventy-eight AR patients were randomly assigned to either the BC99 gummies group (n = 40) or the placebo group (n = 38) for an 8-week intervention. Rhinitis symptom survey questionnaires, including the Total Nasal Symptom Score (TNSS), the Rhinitis Quality of Life Questionnaire (RQLQ), and the Rhinitis Control Assessment Test (RCAT), were administered and evaluated. Additionally, oxidative stress indicators, including superoxide dismutase (SOD), glutathione (GSH), and malondialdehyde (MDA), as well as immune factors (IgA, complement C3) were measured by enzyme-linked immunosorbent assay. Serum metabolomic profiles were analyzed by liquid chromatography-tandem mass spectrometry (LC-MS/MS).

**Results:**

The results indicated that BC99 significantly alleviated nasal symptoms and improved quality of life, as evidenced by reduced TNSS and RQLQ scores (p < 0.05) and increased RCAT scores (p < 0.01). Furthermore, BC99 supplementation significantly decreased serum IgA, complement C3, EOS, and MDA levels (p < 0.05) while enhancing antioxidant capacity (increased GSH, stabilized SOD). Metabolomic analysis revealed that BC99 intervention induced 397 differential metabolite changes (136 upregulated, 261 downregulated) and significantly modulated AR-related metabolic pathways, including biosynthesis of unsaturated fatty acids, phenylalanine metabolism, and arginine biosynthesis (p < 0.05).

**Conclusion:**

This study confirms the efficacy of *W. coagulans* BC99 in ameliorating AR, potentially through immune modulation, oxidative stress reduction, and metabolic pathway regulation. These findings support BC99 as a promising adjunct therapy for AR, though further research is warranted to elucidate its molecular mechanisms and long-term effects.

**Clinical trial registration:**

https://clinicaltrials.gov, identifier NCT06680102.

## 1 Introduction

Allergic rhinitis (AR) is a global health concern associated with substantial economic and societal burdens. In the European Union alone, AR-related productivity losses are estimated to cost nearly 40 billion euros annually (1–3). Epidemiological studies indicate that AR affects approximately 10-30% of adults and up to 40% of children, with prevalence rates continuing to rise (4). AR caused by IgE-mediated reactions to inhaled allergens, and its classical symptoms of AR are nasal itching, sneezing, rhinorrhea, and nasal congestion (5, 6). Disease severity is classified as mild or moderate/severe, while symptom frequency is categorized as intermittent or persistent (7). Current first-line pharmacotherapies for AR include antihistamines, intranasal corticosteroids, and leukotriene receptor antagonists, which effectively alleviate symptoms and suppress allergic inflammation (8). However, allergen-specific immunotherapy (AIT) remains the sole treatment with immunomodulatory potential (9). Despite their efficacy, conventional AR medications are often associated with adverse effects—such as dry mouth, drowsiness, and dizziness—that may significantly impair patients’ quality of life (10). Consequently, there is an urgent need to explore safer and more sustainable therapeutic alternatives.

Probiotics are live microorganisms that confer health benefits by modulating the host’s intestinal microbiota (11). These microorganisms exhibit robust survivability under harsh intestinal conditions, including acidic pH, digestive enzymes, and bile salts. Furthermore, probiotics exert beneficial effects through multiple mechanisms, such as pathogen inhibition and immune system modulation (12). Given these properties, probiotics are increasingly being explored as therapeutic alternatives worldwide. In AR, probiotic supplementation may help modulate immune responses, attenuate reactions to inhaled allergens, and mitigate inflammation-mediated tissue damage. Emerging evidence supports the efficacy of various probiotic strains in AR management, including NVP-1703 (a blend of *Bifidobacterium longum* and *Lactobacillus plantarum*) (13), *Enterococcus faecalis* (14), and specific multi-strain formulations (15). Collectively, these findings highlight the potential of probiotics as a promising strategy for preventing or alleviating allergic diseases, including AR.

*W. coagulans* BC99 is a Gram-positive, anaerobic bacterium with a well-established biosafety profile (16–18). Previous studies have demonstrated its therapeutic potential in various conditions: ameliorating constipation through modulation of sphingolipid metabolism (19), alleviating alcoholism via butyrate-mediated enhancement of intestinal barrier function (20), and reducing hyperuricemia through gut microbiota and metabolic pathway regulation (21). Although many studies have demonstrated the ability of BC99 to modulate gut microbiota, suppress inflammatory responses, and enhance immune function, its efficacy in AR symptoms in humans remains unexplored and warrants further investigation.

Metabolomics is a scientific discipline focused on the systematic study of small-molecule biochemical compounds produced by metabolic pathways in living systems. Due to its high-throughput analytical capabilities, metabolomics has emerged as a powerful tool for identifying diagnostic biomarkers in allergic diseases and evaluating therapeutic interventions. Growing evidence suggests that metabolic dysregulation is closely associated with the pathogenesis of AR (22). Investigating the potential of *W. coagulans* BC99 to alleviate AR symptoms through metabolic modulation is therefore of significant clinical relevance.

In this study, we conducted a randomized, double-blind, placebo-controlled trial to systematically evaluate the effects of an 8-week *W. coagulans* BC99 gummies intervention on AR symptoms, quality of life, immune markers, oxidative stress parameters, and serum metabolic profiles. Maltodextrin was used as the placebo control. Our findings will provide a scientific foundation for the application of *W. coagulans* BC99 in adult AR.

## 2 Materials and methods

### 2.1 Study design

This randomized, double-blind, placebo-controlled study was approved by the Ethics Committee of the First Affiliated Hospital of Henan University of Science and Technology on 25 October 2024 (Approval No. 2024-0496) and registered at https://clinicaltrials.gov (NCT06680102; accessed on 8 November 2024). The study was conducted from October to December 2024 in compliance with the ethical principles of the Declaration of Helsinki. Eligible participants were identified through a screening process involving identity verification and physical examinations. A total of 80 individuals met the inclusion criteria and were enrolled. Before participation, all participants received a thorough explanation of the study procedures and provided written informed consent.

### 2.2 Inclusion/exclusion criteria

Inclusion criteria were as follows: (1) Provision of voluntary written informed consent to participate in the study. (2) Ability to comply with the study protocol requirements. (3) Age between 18 and 65 years. (4) Diagnosis of AR based on the Chinese Guidelines for the Diagnosis and Treatment of AR (2022 Revised Edition). (5) Presence of two or more of the following symptoms: sneezing, watery rhinorrhea, nasal itching, or nasal congestion. Symptoms persisted or accumulated for ≥1 h per day and could be accompanied by ocular symptoms (itching, tearing, or redness). (6) Physical examination findings of pale, edematous nasal mucosa with watery discharge. Only participants fulfilling all the above criteria were enrolled.

The exclusion criteria were as follows: (1) Use of medications affecting gut microbiota (e.g., antibiotics, probiotics, intestinal mucosal protectants, or traditional Chinese herbal preparations) for >1 week within one month prior to screening. (2) Comorbid tuberculosis infection. (3) Concurrent diagnosis of allergic asthma. (4) Presence of nasal polyps or severe deviated nasal septum. (5) History of severe systemic diseases or malignancies. (6) Congenital genetic disorders or immunodeficiency diseases. (7) Regular consumption of probiotics/prebiotics within 6 months before screening. (8) Severe gastrointestinal disorders (e.g., chronic diarrhea, inflammatory bowel disease). (9) Metabolic syndrome (e.g., obesity, dyslipidemia, hypertension, diabetes mellitus). (10) Active sinusitis, otitis media, or respiratory infections. (11) Known hypersensitivity to any probiotic components used in the study. (12) Pregnancy, lactation, or planned pregnancy during the study period. (13) Discontinuation of the trial product or use of additional medications during the study, leading to unevaluable efficacy or incomplete data. (14) Short-term use of functionally similar products interfering with outcome assessment. (15) Inability to complete the trial due to personal circumstances. (16) Investigator-determined unsuitability for participation. Participants meeting any of the above criteria were excluded from the study.

### 2.3 Sample size and randomization

**Figure 1:**
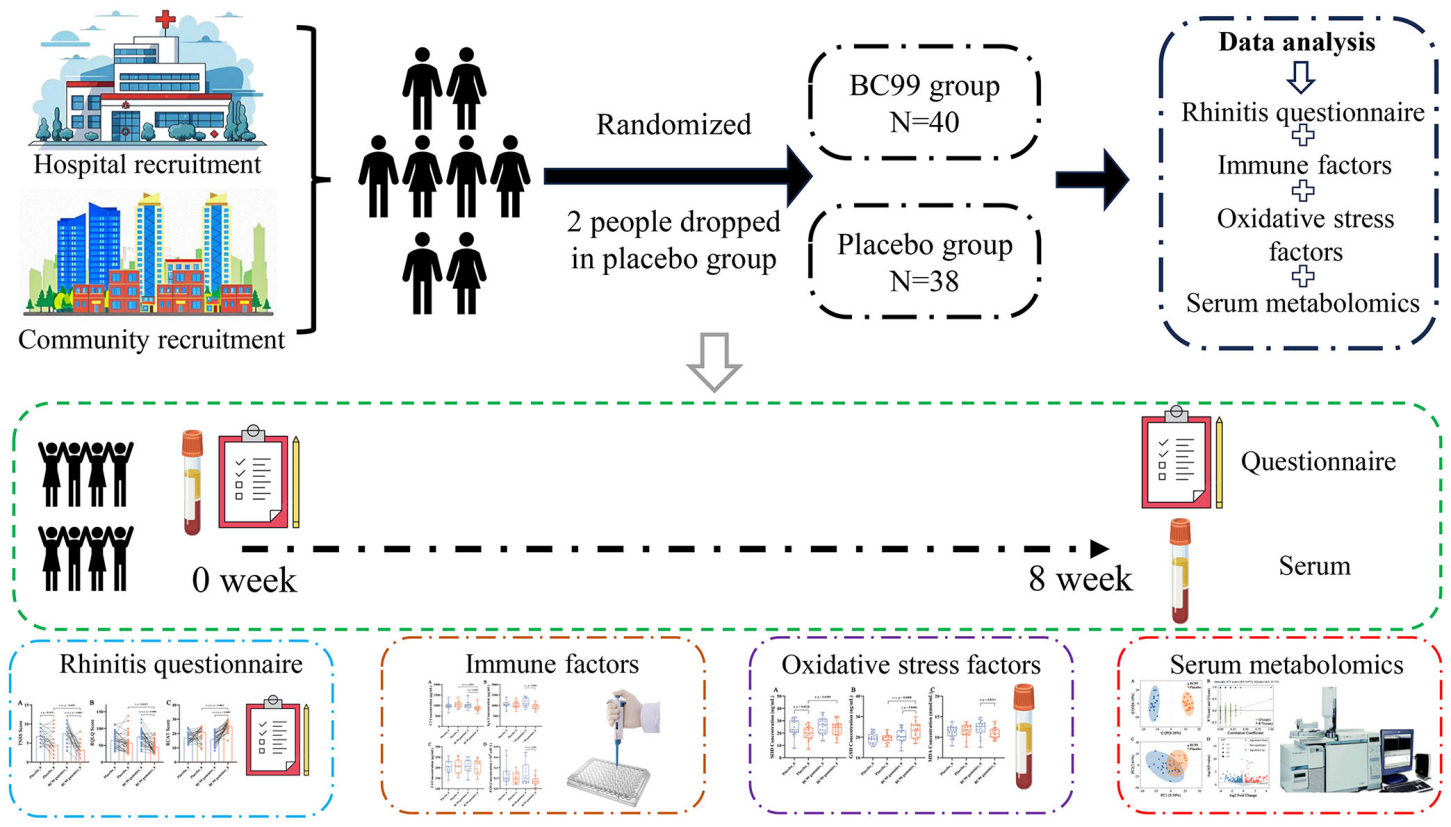
Study flowchart.

Participants were randomly allocated in a 1:1 ratio to either the probiotic group (n=40) or placebo group (n=38) using a computer-generated randomization schedule (**Figure 1**). Randomization was performed with a block size of 4, generating six possible permutation sequences (AABB, ABAB, ABBA, BAAB, BABA, BBAA) to ensure balanced group allocation throughout the study period. The probiotic group received two BC99 probiotic gummies daily (2 × 10^9^ CFU/day; Wecare Probiotics Co., Ltd., Suzhou, China), while the placebo group consumed two identical maltodextrin-based gummies daily for 8 weeks.

### 2.4 Rhinitis symptom survey questionnaire

Survey links and QR codes were distributed to participants to collect the basic demographic information, AR symptom severity, and the impact of symptoms on daily life. Symptom assessment was performed using three validated instruments: the Total Nasal Symptom Score (TNSS) (23), the Rhinitis Quality of Life Questionnaire (RQLQ) (24), and the Rhinitis Control Assessment Test (RCAT) (25). Survey responses were monitored in real time to verify completion, ensuring only valid questionnaires were included in the final analysis.

### 2.5 Blood sample collection and measurement

Venous blood samples were collected from fasting participants at weeks 0 and 8 following standard clinical protocols at Henan University of Science and Technology Hospital. Morning blood samples were centrifuged to obtain serum, which was aliquoted and stored at −80 °C until analysis. Quantitative analysis was performed for immunoglobulin levels (IgA), complement components (C3 and C4), and oxidative stress biomarkers including superoxide dismutase (SOD), glutathione (GSH), and malondialdehyde (MDA). All measurements were conducted using commercially available ELISA kits (Hepeng Biotechnology Co., Ltd., Shanghai, China) strictly following the manufacturer’s protocols, with appropriate quality control samples included in each assay run.

### 2.6 Serum metabolomics analysis

Fasting blood samples were collected from both placebo and BC99 groups after a 12-hour overnight fast at baseline (week 0) and post-intervention (week 8). Serum was isolated by centrifugation at 3000 × g for 15 minutes at 4 °C. For metabolite extraction, 100 μL aliquots of serum were combined with 400 μL of ice-cold extraction solution containing deuterated internal standards. After vortexing for 30 s, samples were sonicated for 10 minutes at 4 °C, followed by protein precipitation at -40 °C for 1 hour. The resulting supernatant was collected after centrifugation at 13,800 × g for 15 minutes at 4 °C and transferred to fresh glass vials for analysis. A pooled quality control (QC) sample was prepared by combining equal volumes of all supernatants.

Chromatographic separation was performed on a Vanquish UHPLC system (Thermo Fisher Scientific, San Jose, CA, USA) equipped with a Waters ACQUITY UPLC BEH Amide column (2.1 × 50 mm, 1.7 μm). The mobile phase consisted of (A) 25 mmol/L ammonium acetate/ammonium hydroxide in water (pH 9.75) and (B) acetonitrile, with the auto-sampler maintained at 4 °C. Mass spectrometric analysis was conducted using an Orbitrap Exploris 120 mass spectrometer (Thermo Fisher Scientific, San Jose, CA, USA) operated in data-dependent acquisition (DDA) mode. The electrospray ionization source parameters included: sheath gas flow (50 Arb), auxiliary gas flow (15 Arb), capillary temperature (320 °C), and spray voltages (± 3.4-3.8 kV). Full MS scans were acquired at 60,000 resolution (m/z 200) with subsequent MS/MS scans at 15,000 resolution using stepped normalized collision energies (20, 30, and 40 eV).

Raw data files were converted to mzXML format using ProteoWizard 3.0 and processed with an in-house R script. Metabolite identification was performed using R packages and BiotreeDB (version 3.0) with a mass accuracy threshold of 5 ppm.

LC-MS raw data were processed through Progenesis QI V2.3 (Nonlinear Dynamics, Newcastle upon Tyne, UK) for baseline correction, peak detection, alignment (retention time correction < 0.5 min), and normalization. Metabolite identification was achieved by matching experimental MS/MS spectra against reference databases (HMDB, LipidMaps V2.3, Metlin) and self-built library, with mass accuracy thresholds of 10 ppm for precursor ions and 20 ppm for fragments. Positive and negative ion mode data were merged into a unified data matrix.

### 2.7 Statistical analysis

Quantitative data were presented as mean ± standard deviation (SD). For normally distributed data, between-group comparisons were analyzed using two-tailed independent samples t-tests, while non-normally distributed data were evaluated with nonparametric Mann-Whitney U tests for inter-group comparisons and Wilcoxon signed-rank tests for intra-group comparisons. Categorical variables expressed as frequencies (percentages) were analyzed using Pearson’s chi-square tests or Fisher’s exact tests. Multivariate statistical analyses were conducted using SIMCA software (version 18.0.1, Sartorius Stedim Data Analytics AB), including principal component analysis (PCA) for unsupervised pattern recognition and orthogonal partial least squares-discriminant analysis (OPLS-DA) for supervised modeling. Metabolic pathway analysis was performed through KEGG pathway database (http://www.genome.jp/kegg/) and MetaboAnalyst platform (http://www.metaboanalyst.ca/), with pathway enrichment of differential metabolites assessed using Fisher’s exact test. A significance threshold of *p* < 0.05 was applied for all statistical tests, with false discovery rate (FDR) correction applied for multiple comparisons where appropriate.

## 3 Results

### 3.1 Baseline characteristics

As presented in **Table 1**, the trial enrolled a total of 78 participants, who were randomly assigned to either the placebo group (n=38) or the probiotics BC99 group (n=40). The BC99 group comprised 21 males and 19 females, with a mean age of 36.50 ± 14.05 years. Similarly, the placebo group included 20 males and 18 females, with a comparable mean age of 37.21 ± 11.96 years. No significant differences in demographic characteristics were observed between the two groups at baseline, ensuring that any subsequent effects could be attributed to the intervention rather than preexisting disparities in age or gender distribution.

**Table 1:** Comparison of general information between the two groups of participants.

Group	Age ( $\bar{x}$ ± *s*)	Sex (%)	
		Man	Women
BC99(n=40)	36.50±14.05	21(52.5%)	19(47.5%)
Placebo(n=38)	37.21±11.96	20(53.0%)	18(47.0%)
*F/x^2^* *P* Value	0.961 0.811	6.770 0.990	

### 3.2 Effects of BC99 gummies on rhinitis symptoms and quality of life in patients with AR

To assess the efficacy of BC99 supplementation in alleviating AR symptoms, the TNSS, RQLQ, and RCAT were measured in both study groups over an 8-week intervention period. The TNSS, a validated AR assessment tool (23), quantifies symptom severity by summing scores across four domains: nasal congestion, rhinorrhea, nasal itching, and sneezing. Each symptom was rated on a 4-point scale (0 = asymptomatic, 1 = mild, 2 = moderate, 3 = severe), with evaluations conducted at baseline and 8 weeks post-intervention. The total TNSS score was used to compare symptom severity before and after probiotic treatment. The RQLQ, a standardized measure of AR-related quality of life, evaluates seven domains, including sleep, mood, and social functioning (24). Responses were scored from 0 (no impairment) to 6 (severe impairment), with the overall score calculated as the mean across all items (range: 0–6). Lower scores indicate better quality of life. The intervention’s impact on patients’ well-being was quantified by analyzing changes between baseline and week 8. Additionally, the RCAT, a six-item questionnaire (25), assessed rhinitis control by evaluating nasal congestion, sneezing, tearing, sleep disturbances, daily activities, and overall symptom management. Each item was scored from 1 to 5, with higher cumulative scores reflecting better disease control.

**Figure 2:**
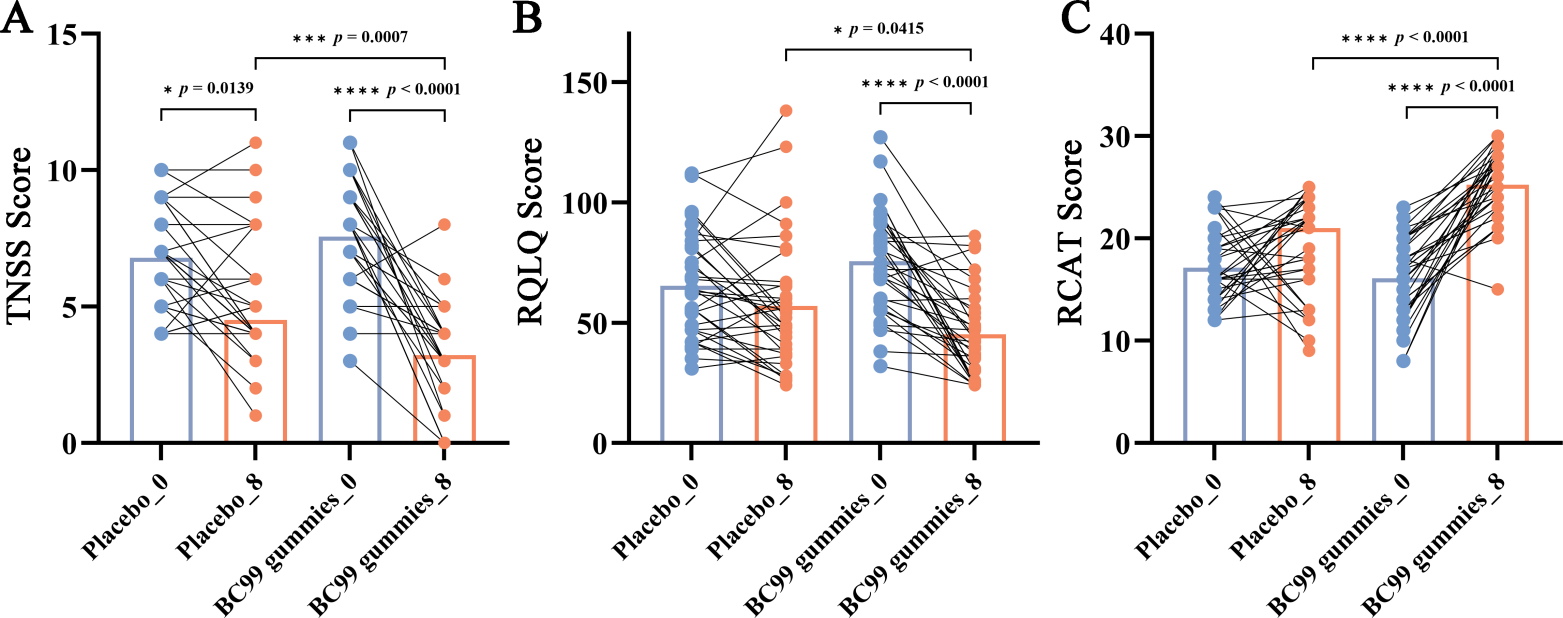
Changes in TNSS, RQLQ, and RCAT scores following BC99 gummy intervention. **(A)** TNSS scores in the placebo and BC99 groups. **(B)** RQLQ scores in the placebo and BC99 groups. **(C)** RCAT scores in the placebo and BC99 groups. * *p* < 0.05, *** *p* < 0.001, **** *p* < 0.0001.

As shown in **Figure 2**, in the BC99 group, after the 8-week intervention period, the mean TNSS score significantly decreased from 7.57 to 3.21, the mean RQLQ score significantly decreased from 75.50 to 45.15, and the mean RCAT score significantly increased from 16.09 to 25.26 (*p* < 0.05 for all). Compared with the placebo group, the TNSS and RQLQ in the BC99 gummies group were significantly reduced (*p* < 0.05), while the RCAT score was significantly increased (*p* < 0.0001), indicating that BC99 gummies intervention can help alleviate AR symptoms and improve quality of life.

### 3.3 Effects of BC99 gummies on immune factors and eosinophil levels in AR patients

To evaluate the immunomodulatory effects of BC99 supplementation, serum levels of immune factors (IgA, C3, and C4) and eosinophil (EOS) counts were measured in AR patients before and after the 8-week intervention period.

**Figure 3:**
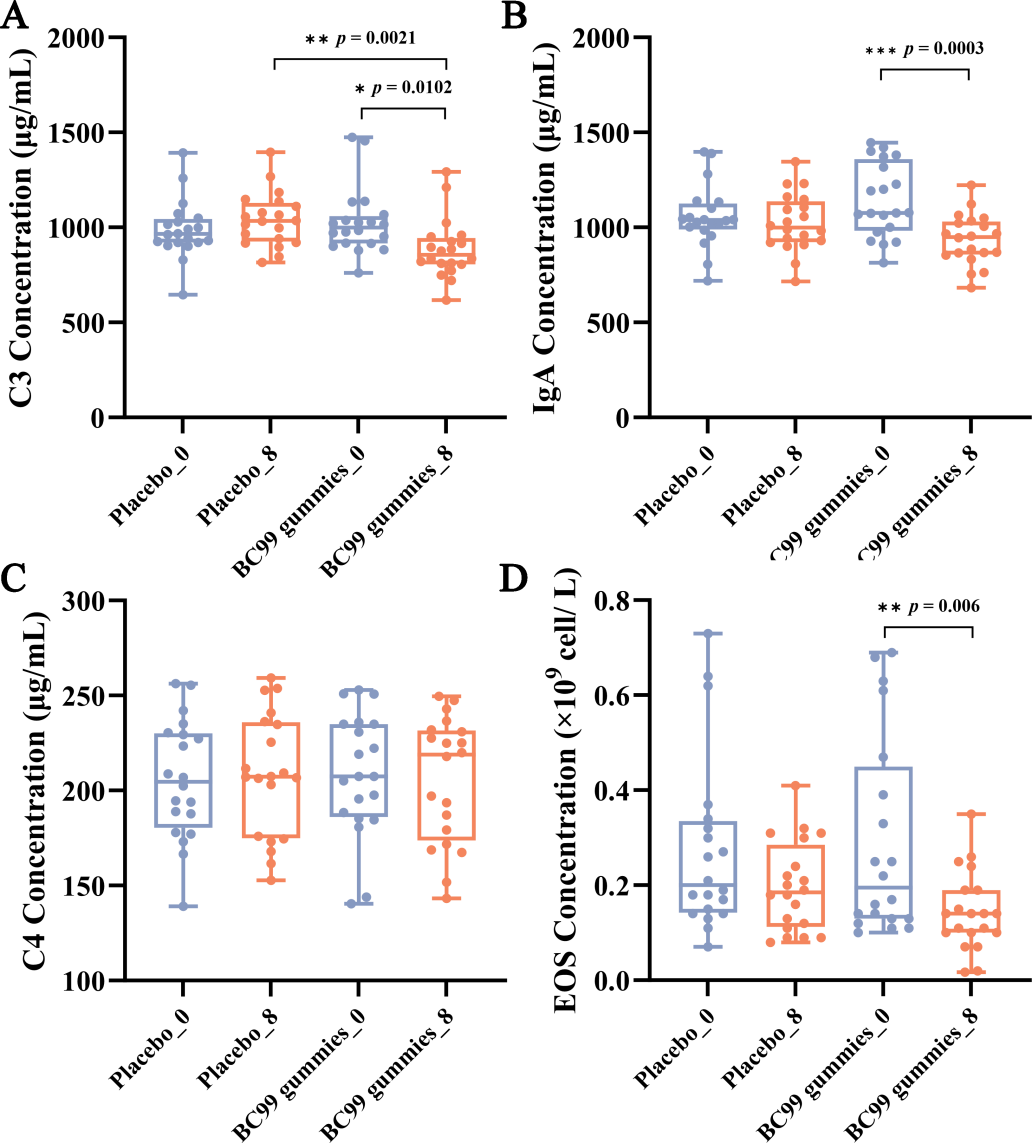
Effect of BC99 intervention on immune factor levels in participants with AR. **(A)** C3 levels. **(B)** IgA levels. **(C)** C4 levels. **(D)** EOS counts. * *p* < 0.05, ** *p* < 0.01, *** *p* < 0.001.

No significant differences were observed between the placebo group and the BC99 group at baseline. The placebo group showed no significant changes before and after intervention. While after intervention in the BC99 group, C3 levels decreased from 1026.50 μg/mL to 885.45 μg/mL (*p* = 0.0102), IgA levels decreased from 1143.45 μg/mL to 936.30 μg/mL (*p* = 0.0003), and EOS counts decreased from 0.21 × 10^9/^L to 0.08 × 10^9/^L (*p* = 0.006) (**Figures 3A, B, D**). In contrast, complement C4 levels remained unchanged, showing no statistically significant difference between baseline and post-treatment measurements (**Figure 3C**). These findings suggest that BC99 gummies may modulate specific immune parameters while maintaining stability in others.

### 3.4 Effects of BC99 gummies intervention on oxidative stress in AR patients

**Figure 4:**
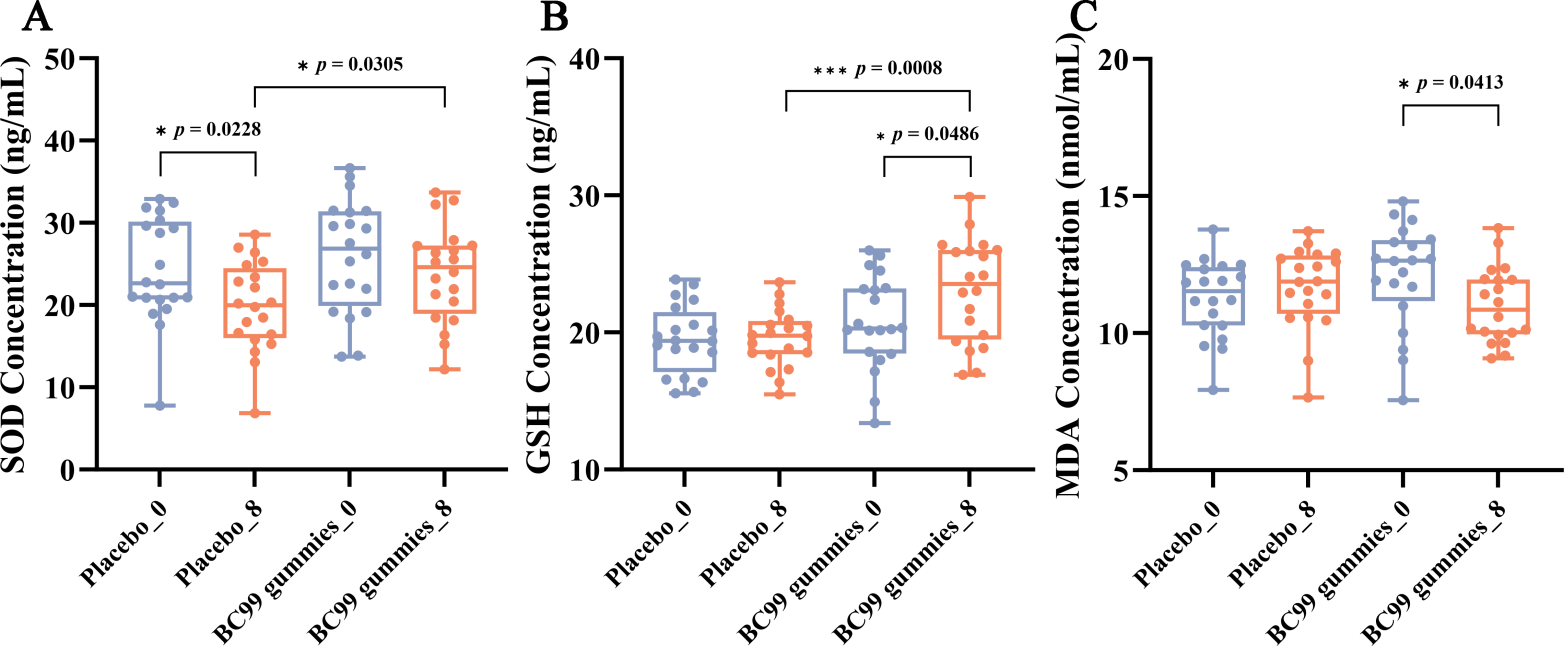
Effect of BC99 intervention on oxidative stress markers in participants with AR. **(A)** SOD activity. **(B)** GSH levels. **(C)** MDA levels. * *p* < 0.05, *** *p* < 0.001.

SOD activity serves as a key indicator of the body’s antioxidant capacity. In this study, comparative analysis of SOD levels between the placebo group and the BC99 gummies group revealed a significant decline in the placebo group after 8 weeks of intervention (*p* = 0.0228), whereas the BC99 group maintained stable SOD levels (**Figure 4A**). These results demonstrate that BC99 gummies intervention effectively preserves SOD activity in AR patients. GSH, a critical endogenous free radical scavenger, is essential for maintaining oxidative balance (26). The post-intervention data revealed that compared with baseline, the BC99 group exhibited a significant increase in GSH levels from 20.76 ng/mL to 23.07 ng/mL (*p* = 0.0486, **Figure 4B**), suggesting that BC99 gummies positively modulate the oxidation/antioxidant system. Furthermore, MDA, a marker of lipid peroxidation, reflects oxidative stress severity. Elevated MDA levels compromise biological membrane integrity and impair physiological functions (27). As depicted in **Figure 4C**, the BC99 group exhibited a significant decrease in MDA levels from12.11 at baseline to 11.01 post-intervention (*p* = 0.0413), indicating that probiotic supplementation mitigates oxidative stress-induced lipid peroxidation.

### 3.5 Effects of BC99 gummies intervention on serum metabolic profile of AR patients

**Figure 5:**
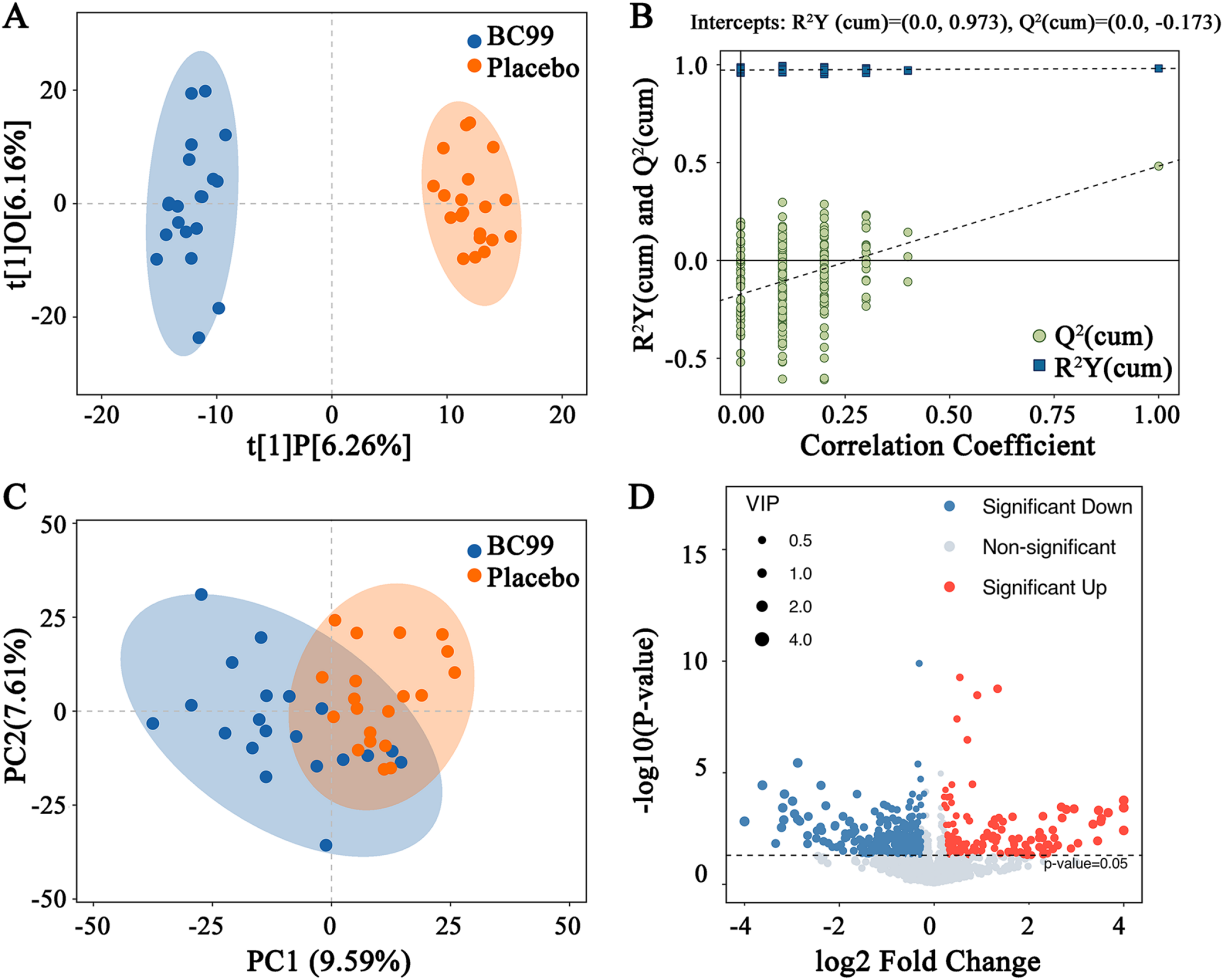
Serum metabolic profile changes in participants with AR after 8 weeks of BC99 intervention. **(A)** OPLS-DA score plot. **(B)** OPLS-DA permutation test. **(C)** PCA score plot. **(D)** Volcano plot visualizing differential metabolites (red: significantly upregulated, blue: significantly downregulated, gray: non-significant changes).

To investigate the regulatory effects of BC99 gummies on serum metabolites, we compared metabolic profiles between the BC99 and placebo groups using multivariate statistical analysis. OPLS-DA revealed a clear separation between the two groups, indicating distinct metabolic patterns (VIP > 1). The permutation test confirmed model validity (R² Y = 0.973, Q² = −0.173), with no overfitting observed (**Figures 5A, B**). PCA further demonstrated metabolic profile differences, with the first three principal components accounting for 22.97% of total variance (**Figure 5C**). Statistical analysis identified significant alterations in metabolite regulation following BC99 intervention. Compared to the placebo group, the BC99 group exhibited 136 significantly upregulated and 261 significantly downregulated serum metabolites (**Figure 5D**). **Supplementary Table S1** contains detailed information of serum metabolites between the BC99 and placebo groups after 8-week intervention.

**Figure 6:**
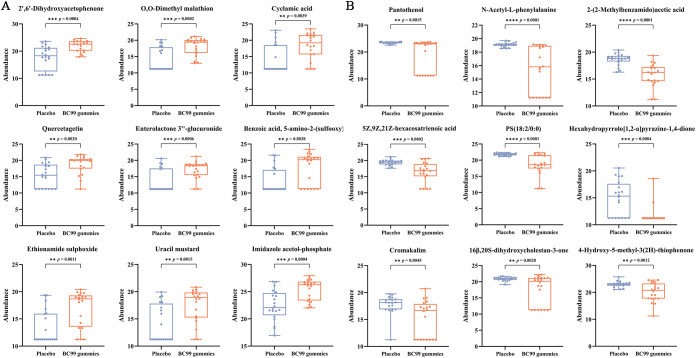
Alterations in serum metabolite levels in AR participants following 8-week BC99 intervention. **(A)** Relative abundance of upregulated metabolites. **(B)** Relative abundance of downregulated metabolites. ** *p* < 0.01, *** *p* < 0.001, **** *p* < 0.0001.

Further analysis of the top 10 differentially expressed metabolites classified them into eight categories: vitamins and derivatives, amino acids and derivatives, lipids, organic acids and derivatives, heterocyclic compounds, steroids, phenols and aromatic compounds, and endogenous metabolites and conjugates. Notably, BC99 intervention led to significant upregulation (*p* < 0.05) of metabolites such as enterolactone-3’’-glucuronide, 2’,6’-dihydroxyacetophenone, and quercetin (phenolic and endogenous metabolites) (**Figure 6A**). Conversely, pantothenol, N-Acetyl-L-phenylalanine, 2-(2-methylbenzoylamino) acetic acid, PS (18:2/0:0), 5Z,9Z,21Z-hexacosatrienoic acid, and 16β,20S-dihydroxycholestane-3-one (vitamins, amino acids, lipid and steroids-related compounds) were significantly downregulated (*p* < 0.05) (**Figure 6B**). These findings demonstrate that BC99 gummies significantly modulate serum metabolism in AR patients, particularly affecting pathways involving vitamins, lipids, and phenolic compounds.

### 3.6 KEGG classification and enrichment analysis of differential metabolites

**Figure 7:**
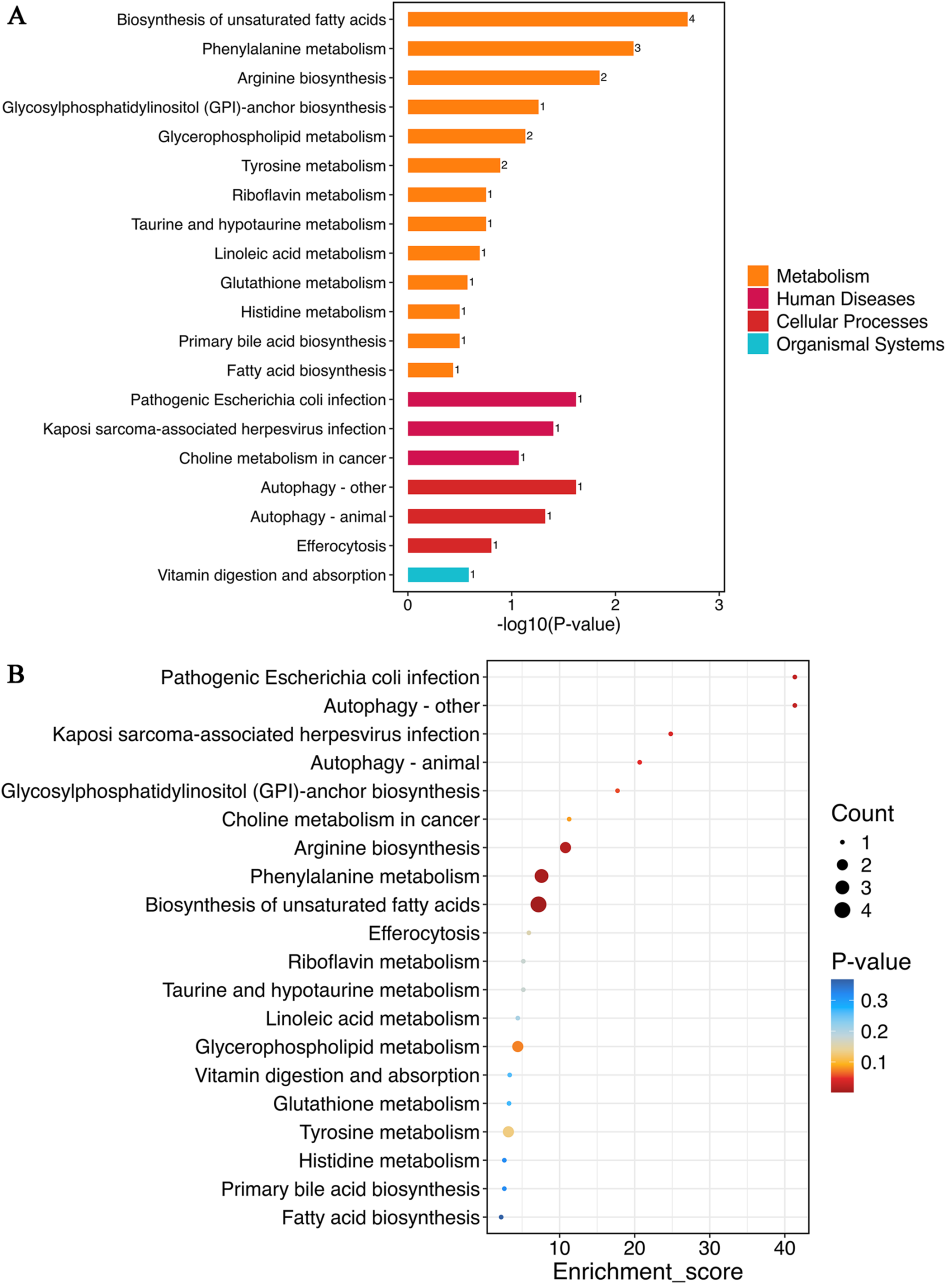
Metabolic pathway analysis of differential metabolites between placebo and BC99 groups. **(A)** KEGG functional classification. The numbers on the bars represent the count of differential metabolites annotated to the pathway. **(B)** KEGG enrichment analysis.

Metabolic pathway analysis of serum differential metabolites (VIP > 1, *p* < 0.05) was performed using MetaboAnalyst 5.0 to compare the placebo and BC99 gummies groups. **Figure 7A** presents the top 20 most significantly KEGG pathways (the smallest *p*-values). Metabolomic analysis demonstrated that BC99 intervention induced significant alterations (p<0.05) in 13 metabolites spanning 7 KEGG pathways, particularly affecting the biosynthesis of unsaturated fatty acids, phenylalanine metabolism, arginine biosynthesis, autophagy – other, pathogenic *Escherichia coli* infection, Kaposi sarcoma-associated herpesvirus infection, and autophagy – animal. Notably, amino acid metabolism pathways contained the most significantly differential metabolites, with phenylalanine metabolism encompassing 3 metabolites and arginine biosynthesis comprising 2 metabolites. Within lipid metabolism, biosynthesis of unsaturated fatty acids involved 4 differential metabolites.

**Figure 7B** displays the KEGG enrichment analysis of the top 20 most significant pathways (smallest *p*-values). These pathways, including biosynthesis of unsaturated fatty acids, phenylalanine metabolism, and arginine biosynthesis, exhibited both the highest metabolite counts and the lowest *p*-values. These results demonstrate that BC99 gummies intervention predominantly affects lipid and amino acid-related metabolic pathways in AR patients.

### 3.7 Correlation analysis between key metabolites and AR related indicators

**Figure 8:**
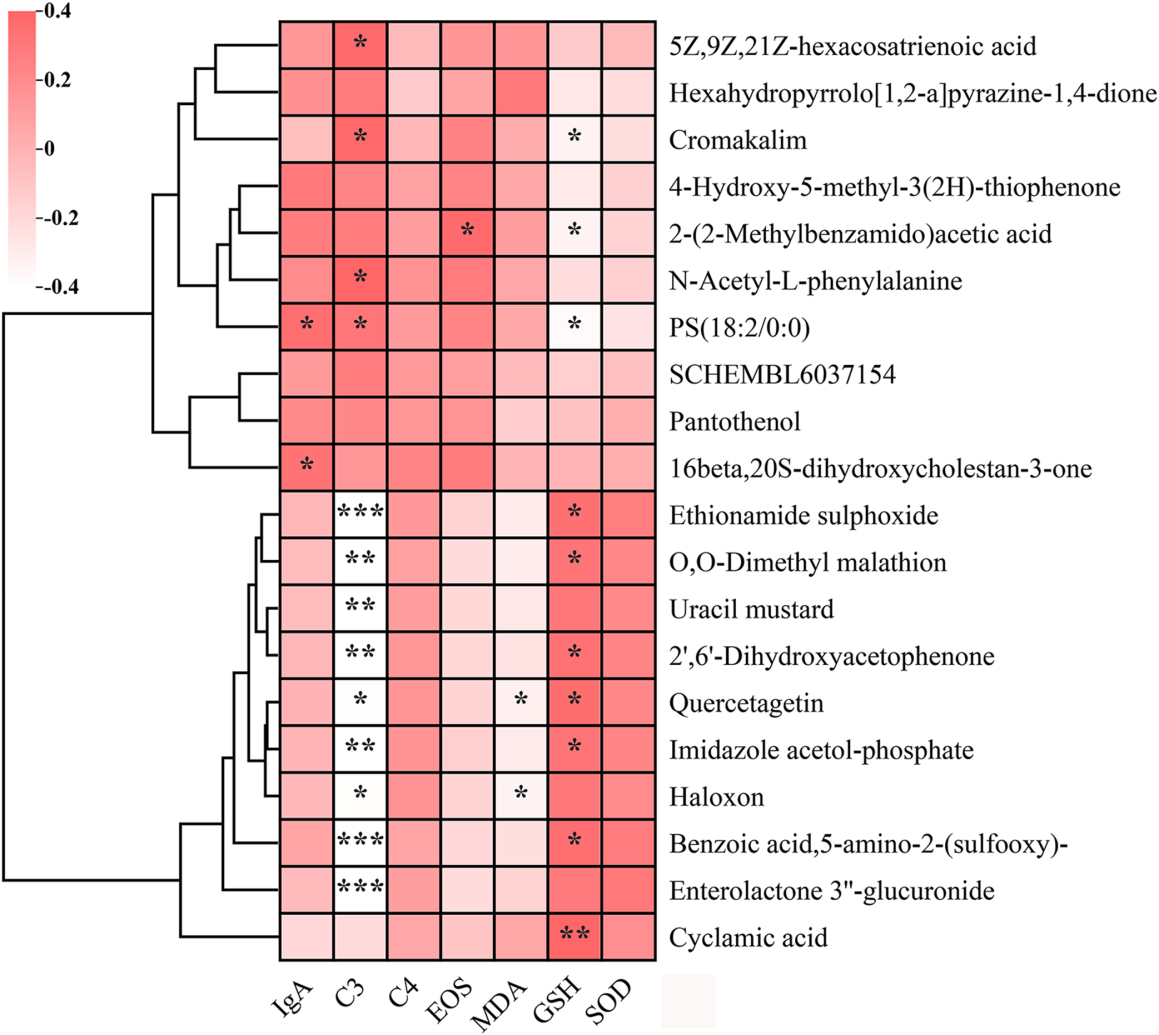
Correlation analysis between key serum metabolites and immune/oxidative stress markers in placebo and BC99 groups. Pink and white indicate positive and negative correlations, respectively (*p < 0.05, **p < 0.01, ***p < 0.001).

To investigate potential associations between key serum metabolites and AR-related biomarkers, we conducted correlation analysis (**Figure 8**). The analysis incorporated immunological factors, oxidative stress markers, and the top 10 most significantly upregulated and downregulated metabolites. Notably, several metabolites - including ethionamide sulphoxide, benzoic acid (5-amino-2-(sulfooxy)-), and enterolactone 3’’-glucuronide - exhibited significant negative correlations with complement C3 levels (*p* < 0.001). Following BC99 intervention, cyclamic acid, quercetagetin, ethionamide sulphoxide, and benzoic acid (5-amino-2-(sulfooxy)-) showed significant positive correlations with GSH levels (*p* < 0.05), indicating a potential regulatory role in oxidative stress modulation. Additionally, quercetagetin and haloxon demonstrated a significant inverse relationship with MDA levels (*p* < 0.05).

Of particular interest, imidazole acetol-phosphate, a key intermediate in histidine biosynthesis, displayed dual associations: a negative correlation with complement C3 (*p* < 0.05) and a positive correlation with GSH (*p* < 0.05). As this metabolite participates in imidazole ring modification and amino group integration within the histidine biosynthesis pathway, our findings suggest that differential serum metabolites may influence AR clinical manifestations through histidine metabolism-mediated regulatory mechanisms.

## 4 Discussion

AR is a prevalent global disease. With increasing quality of life and heightened health awareness, identifying safe and effective dietary interventions for AR prevention and symptom alleviation has become a critical long-term objective. Probiotics have emerged as a promising therapeutic option due to their potential to modulate immune responses, reduce inflammation, and restore intestinal microbiota balance (28, 29). However, the underlying mechanisms remain incompletely understood. This study investigated the effects of an 8-week BC99 gummy intervention on biomarkers and metabolic profiles associated with AR remission. No significant differences were observed in hematological parameters between groups before and after treatment, and no adverse reactions were reported, confirming the safety of BC99 gummies.

This study demonstrates that BC99 gummy supplementation effectively improves nasal symptoms, reduces rhinitis severity, and enhances quality of life in patients with AR. These benefits are supported by significant reductions in TNSS and RQLQ scores, along with increased RCAT scores. The therapeutic effects of BC99 may be attributed to its ability to modulate key immune pathways involved in AR pathogenesis. AR is characterized by dysregulated immune responses, including IgA-mediated inflammation. Under pathological conditions such as structural abnormalities, immune complex deposition, or chronic inflammation, IgA promotes inflammatory cell activation via complement activation and Fcα receptor (FcαR) signaling. This process contributes to tissue damage and exacerbates AR symptoms (30). Additionally, the C3 plays a central role in AR progression. Cleavage of C3 into C3a and C3b fragments activates inflammatory cells, disrupts the mucosal barrier, and amplifies T-helper 2 (Th2) immune responses, further aggravating AR symptoms. EOS, the primary effector cells in Th2-mediated inflammation, are recruited to the nasal mucosa by cytokines such as interleukin-5 (IL-5). Upon activation, EOS release cytotoxic proteins, including eosinophil cationic protein (ECP) and major basic protein (MBP), leading to epithelial damage, mucus hypersecretion, and worsening of nasal inflammation. These pathological changes manifest clinically as nasal congestion, rhinorrhea, and other AR symptoms (31). BC99 may exert its beneficial effects by regulating immune-related cytokines such as IgA and C3, thereby improving intestinal health and metabolic function.

Emerging evidence indicates that IgA plays a significant role in EOS activation and degranulation (32–34). Mechanistically, IgA binds to the Fcα receptor (FcαR/CD89) on EOS surfaces, triggering intracellular signaling cascades involving Syk kinase and the PI3K pathway (35). This interaction induces EOS to release pre-synthesized cytotoxic granule proteins, including MBP, ECP, and eosinophil peroxidase (EPO), which directly damage tissues and perpetuate inflammatory responses. Following degranulation, EOS further amplify inflammation through chemokine release. Key mediators such as C-C motif chemokine ligand 5 (CCL5/RANTES) and CCL11 (eotaxin-1) promote bone marrow and circulating EOS migration to inflammatory sites, establishing a self-reinforcing cycle of inflammation (36). Notably, our intervention with BC99 gummies significantly reduced serum levels of IgA, complement component C3, and EOS (p<0.05). These findings demonstrate BC99’s capacity to attenuate multiple pathological pathways in allergic rhinitis (AR), including: IgA-mediated EOS activation and degranulation, Complement system activation via C3 reduction, and inflammatory cell recruitment through EOS suppression. The simultaneous downregulation of these biomarkers strongly suggests that BC99 exerts its therapeutic effects by interrupting critical nodes in the AR inflammatory cascade. This multi-target mechanism distinguishes BC99 from single-pathway inhibitors and may explain its clinical efficacy in improving AR symptoms.

Oxidative stress is implicated in the pathogenesis of various diseases, including cardiovascular and neurodegenerative disorders (37, 38). As a critical antioxidant in the human body, GSH protects nasal mucosal epithelial cells from oxidative damage by scavenging free radicals such as reactive oxygen species (ROS) (26). Following intervention with BC99 gummies, the GSH content increased significantly (*p* < 0.05), suggesting enhanced antioxidant activity. Conversely, MDA, the end product of lipid peroxidation, serves as a biomarker of oxidative stress. Elevated MDA levels may exacerbate nasal mucosal inflammation by damaging epithelial cells and activating the NF-κB pathway (27). Notably, after BC99 gummies intervention, MDA levels decreased significantly (*p* < 0.05), indicating reduced oxidative damage. SOD, a key antioxidant enzyme, catalyzes the conversion of superoxide radicals into oxygen and hydrogen peroxide, thereby mitigating ROS accumulation and cellular oxidative stress (39). While SOD levels remained stable in the BC99 group, they declined significantly in the placebo group (*p* < 0.05), further supporting the protective role of BC99 gummies. Collectively, these findings suggest that BC99 gummies may alleviate oxidative stress-induced nasal mucosal damage by enhancing systemic antioxidant capacity, providing a molecular basis for ameliorating AR symptoms. This observation aligns with previous studies demonstrating that probiotics can modulate the gut-microbiota-immune axis, suppress eosinophil infiltration, and reduce free radical production, thereby improving AR pathology (40).

To further elucidate the underlying mechanism by which BC99 gummies alleviate AR, serum metabolomic analysis was conducted in both the placebo and BC99 groups to assess metabolic changes at week 8. The analysis revealed that BC99 intervention significantly altered the metabolic profile of AR patients. Specifically, BC99 administration modulated 397 serum metabolites compared to the placebo group, with 136 metabolites significantly upregulated and 261 significantly downregulated. Among these, 20 metabolites were identified as key biomarkers. Notably, four differential metabolites—imidazole acetol-phosphate, quercetagetin, 5-amino-2-(sulfooxy)-Benzoic acid, and enterolactone 3’’-glucuronide—exhibited substantial increases following BC99 intervention. Additionally, KEGG enrichment analysis indicated that BC99 supplementation significantly influenced metabolic pathways, including biosynthesis of unsaturated fatty acids, phenylalanine metabolism, and arginine biosynthesis.

Furthermore, we examined the correlation between key differential metabolites and clinical indicators of AR. As illustrated in the **Figure 8**, imidazole acetol-phosphate—a metabolite associated with histidine metabolism—demonstrated a significant negative correlation with complement C3 and a positive correlation with GSH. These findings indicate that the BC99-mediated alleviation of AR symptoms may be mechanistically linked to modulations in histidine metabolism.

Emerging evidence suggests a potential link between AR and histidine metabolism (41). As an essential amino acid, histidine yields various metabolites—including histamine and urocanic acid—that play crucial roles in immune regulation and inflammatory responses, particularly in maintaining mucosal barrier integrity and mediating allergic signaling (41, 42). Disruptions in histidine metabolism may contribute to AR pathogenesis through multiple mechanisms. First, histamine—produced via histidine decarboxylase (HDC)-mediated catalysis—binds to H1 receptors on mast cells, triggering classic AR symptoms (e.g., nasal pruritus and sneezing) while promoting Th2 cytokine secretion (e.g., IL-4, IL-13) and eosinophil infiltration (43). Second, urocanic acid, a histidine metabolic intermediate, modulates dendritic cell (DC) antigen presentation, thereby influencing immune tolerance. An imbalance in urocanic acid dynamics may elevate allergen-specific IgE levels. Furthermore, diminished histamine activity can perturb downstream processes, including inflammatory cascades and cellular signaling, ultimately redirecting metabolic flux through the histidine pathway (41).

The intestinal and nasal microbiota also appear to regulate histidine metabolism in AR pathogenesis. Under homeostatic conditions, commensal bacteria metabolize histidine into anti-inflammatory compounds that suppress NF-κB activation, thereby mitigating nasal mucosal inflammation (44, 45). In AR patients, microbial dysbiosis - characterized by diminished probiotic populations and expansion of opportunistic pathogens like Staphylococcus - may exacerbate histamine-driven allergic responses through competitive histidine utilization and pro-inflammatory metabolite production (46). Critical knowledge gaps remain regarding: (1) the mechanistic interplay between histidine metabolic pathways and the nasal/intestinal microenvironment, and (2) the therapeutic potential of targeting specific microbial metabolites for AR intervention. Future studies should address these aspects to advance our understanding of microbial-host metabolic crosstalk in allergic inflammation.

## 5 Conclusion

This clinical study demonstrates that an 8-week intervention with *W. coagulans* BC99 significantly alleviated AR symptoms and improved patients’ quality of life. The probiotic treatment effectively reduced key markers of allergic immune response, including immune factors (IgA, complement C3), EOS indicators, and oxidative stress markers (MDA). Metabolomic analysis revealed that BC99 intervention altered 397 serum metabolites and significantly modulated AR-related metabolic pathways, particularly biosynthesis of unsaturated fatty acids, phenylalanine metabolism, and arginine biosynthesis. Notably, correlation analyses identified BC99-induced metabolic changes - such as increased imidazole acetol-phosphate levels - that showed negative associations with pro-inflammatory markers (e.g., C3) and positive correlations with antioxidant indicators (e.g., GSH). These findings suggest BC99 mediates its therapeutic effects through histidine-dependent immunometabolic crosstalk. Our results establish *W. coagulans* BC99 as an effective intervention for AR management and provide a scientific foundation for its clinical application. Future studies should further elucidate the molecular mechanisms underlying BC99’s metabolic and immunoregulatory effects on AR pathogenesis.

## Data Availability

The original contributions presented in the study are included in the article/**Supplementary Material**. Further inquiries can be directed to the corresponding authors.

## References

[1] Vandenplas O Vinnikov D Blanc PD Agache I Bachert C Bewick M. Impact of rhinitis on work productivity: A systematic review. J Allergy Clin Immunol Pract. (2018) 6:1274–86.e9. doi: 10.1016/j.jaip.2017.09.002, PMID: 29017832

[2] Devillier P Bousquet J Salvator H Naline E Grassin-Delyle S de Beaumont O. In allergic rhinitis, work, classroom and activity impairments are weakly related to other outcome measures. Clin Exp Allergy. (2016) 46:1456–64. doi: 10.1111/cea.12801, PMID: 27562177

[3] Zuberbier T Lötvall J Simoens S Subramanian SV Church MK. Economic burden of inadequate management of allergic diseases in the European union: A ga2len review. Allergy. (2014) 69:1275–9. doi: 10.1111/all.12470, PMID: 24965386

[4] Liu Y Liu Z. Epidemiology, prevention and clinical treatment of allergic rhinitis: more understanding, better patient care. J Clin Med. (2022) 11:6062. doi: 10.3390/jcm11206062, PMID: 36294381 PMC9605427

[5] Bousquet J Schünemann HJ Sousa-Pinto B Zuberbier T Togias A Samolinski B. Concepts for the development of person-centered, digitally enabled, artificial intelligence—Assisted aria care pathways (Aria 2024). J Allergy Clin Immunol: In Pract. (2024) 12:2648–68.e2. doi: 10.1016/j.jaip.2024.06.040, PMID: 38971567

[6] Brożek JL Bousquet J Agache I Agarwal A Bachert C Bosnic-Anticevich S. Allergic rhinitis and its impact on asthma (Aria) guidelines—2016 revision. J Allergy Clin Immunol. (2017) 140:950–8. doi: 10.1016/j.jaci.2017.03.050, PMID: 28602936

[7] Bousquet J van Cauwenberge P Khaltaev N. Allergic rhinitis and its impact on asthma. J Allergy Clin Immunol. (2001) 108:S147–334. doi: 10.1067/mai.2001.118891, PMID: 11707753

[8] Bousquet J Khaltaev N Cruz AA Denburg J Fokkens WJ Togias A. Allergic rhinitis and its impact on asthma (Aria) 2008. Allergy. (2008) 63:8–160. doi: 10.1111/j.1398-9995.2007.01620.x, PMID: 18331513

[9] Wilson DR Torres Lima M Durham SR. Sublingual immunotherapy for allergic rhinitis: systematic review and meta-analysis. Allergy. (2005) 60:4–12. doi: 10.1111/j.1398-9995.2005.00699.x, PMID: 15575924

[10] Platt M. Pharmacotherapy for allergic rhinitis. Int Forum Allergy Rhinol. (2014) 4:S35–40. doi: 10.1002/alr.21381, PMID: 25182353

[11] George Kerry R Patra JK Gouda S Park Y Shin H-S Das G. Benefaction of probiotics for human health: A review. J Food Drug Anal. (2018) 26:927–39. doi: 10.1016/j.jfda.2018.01.002, PMID: 29976412 PMC9303019

[12] Maslowski KM Mackay CR. Diet, gut microbiota and immune responses. Nat Immunol. (2011) 12:5–9. doi: 10.1038/ni0111-5, PMID: 21169997

[13] Kang MG Han SW Kang HR Hong SJ Kim DH Choi JH. Probiotic nvp-1703 alleviates allergic rhinitis by inducing il-10 expression: A four-week clinical trial. Nutrients. (2020) 12:1427. doi: 10.3390/nu12051427, PMID: 32429063 PMC7284371

[14] Schaefer M Enck P. Effects of a probiotic treatment (Enterococcus faecalis) and open-label placebo on symptoms of allergic rhinitis: study protocol for a randomised controlled trial. BMJ Open. (2019) 9:e031339. doi: 10.1136/bmjopen-2019-031339, PMID: 31662387 PMC6830672

[15] Lungaro L Malfa P Manza F Costanzini A Valentini G Squarzanti DF. Clinical efficacy of probiotics for allergic rhinitis: results of an exploratory randomized controlled trial. Nutrients. (2024) 16:4173. doi: 10.3390/nu16234173, PMID: 39683566 PMC11644003

[16] Makizaki Y Uemoto T Yokota H Yamamoto M Tanaka Y Ohno H. Improvement of loperamide-induced slow transit constipation by bifidobacterium bifidum G9–1 is mediated by the correction of butyrate production and neurotransmitter profile due to improvement in dysbiosis. PloS One. (2021) 16:e0248584. doi: 10.1371/journal.pone.0248584, PMID: 33750988 PMC7984621

[17] Konuray G Erginkaya Z. Potential use of bacillus coagulans in the food industry. Foods. (2018) 7:92. doi: 10.3390/foods7060092, PMID: 29899254 PMC6025323

[18] Zhu M Zhu J Fang S Zhao B. Complete genome sequence of heyndrickxia (Bacillus) coagulans bc99 isolated from a fecal sample of a healthy infant. Microbiol Resource Announcements. (2023) 13:e00449–23. doi: 10.1128/mra.00449-23, PMID: 38095439 PMC10793334

[19] Fan Q Gao Y Zhou Y Wu J Wang H Dong Y. Weizmannia coagulans bc99 relieves constipation symptoms by regulating inflammatory, neurotransmitter, and lipid metabolic pathways: A randomized, double-blind, placebo-controlled trial. Foods. (2025) 14:654. doi: 10.3390/foods14040654, PMID: 40002098 PMC11854163

[20] Li C Zhai S Duan M Cao L Zhang J Wang Y. Weizmannia coagulans bc99 enhances intestinal barrier function by modulating butyrate formation to alleviate acute alcohol intoxication in rats. Nutrients. (2024) 16:4142. doi: 10.3390/nu16234142, PMID: 39683538 PMC11643948

[21] Gao Y Li C Li J Duan M Li X Zhao L. Weizmannia coagulans bc99 alleviates hyperuricemia and oxidative stress via daf-16/skn-1 activation in caenorhabditis elegan. Front Microbiol. (2024) 15:1498540. doi: 10.3389/fmicb.2024.1498540, PMID: 39723130 PMC11668962

[22] Liu QD Pan GX Yan YJ Li JW Zhang JJ Liu HL. Metabolomic profiles in allergic rhinitis: A systematic review and meta-analysis. Ann Allergy Asthma Immunol. (2025) 134:594–602.e2. doi: 10.1016/j.anai.2024.12.022, PMID: 39824455

[23] Downie SR Andersson M Rimmer J Leuppi JD Xuan W Akerlund A. Symptoms of persistent allergic rhinitis during a full calendar year in house dust mite-sensitive subjects. Allergy. (2004) 59:406–14. doi: 10.1111/j.1398-9995.2003.00420.x, PMID: 15005764

[24] Juniper EF Thompson AK Ferrie PJ Roberts JN. Validation of the standardized version of the rhinoconjunctivitis quality of life questionnaire. J Allergy Clin Immunol. (1999) 104:364–9. doi: 10.1016/S0091-6749(99)70380-5, PMID: 10452758

[25] Liedtke JP Mandl A Köther J Chwieralski J Shah-Hosseini K Raskopf E. Rcat reflects symptom control and quality of life in allergic rhinoconjunctivitis patients. Allergy. (2018) 73:1101–9. doi: 10.1111/all.13362, PMID: 29159975

[26] Zaknun D Schroecksnadel S Kurz K Fuchs D. Potential role of antioxidant food supplements, preservatives and colorants in the pathogenesis of allergy and asthma. Int Arch Allergy Immunol. (2011) 157:113–24. doi: 10.1159/000329137, PMID: 21986480

[27] Wang Q Miller DJ Bowman ER Nagarkar DR Schneider D Zhao Y. Mda5 and tlr3 initiate pro-inflammatory signaling pathways leading to rhinovirus-induced airways inflammation and hyperresponsiveness. PloS Pathog. (2011) 7:e1002070. doi: 10.1371/journal.ppat.1002070, PMID: 21637773 PMC3102730

[28] Wang J Bai X Peng C Yu Z Li B Zhang W. Fermented milk containing lactobacillus casei zhang and bifidobacterium animalis ssp. Lactis V9 alleviated constipation symptoms through regulation of intestinal microbiota, inflammation, and metabolic pathways. J Dairy Sci. (2020) 103:11025–38. doi: 10.3168/jds.2020-18639, PMID: 33222846

[29] Park S-A Lee G-H Hoang T-H Lee H-Y Kang I-Y Chung M-J. Heat-inactivated lactobacillus plantarum nf1 promotes intestinal health in loperamide-induced constipation rats. PloS One. (2021) 16:e0250354. doi: 10.1371/journal.pone.0250354, PMID: 33872333 PMC8055018

[30] Zheng H Xu S Yang R Jiao W-E Qiao Y-L Liu J-Y. Changes in and potential mechanisms of circulating iga+Cd27-class-switched memory B cells in patients with allergic rhinitis. J Asthma Allergy. (2025) 18:69–83. doi: 10.2147/jaa.s501775, PMID: 39867643 PMC11766316

[31] Lim MC Taylor RM Naclerio RM. The histology of allergic rhinitis and its comparison to cellular changes in nasal lavage. Am J Respir Crit Care Med. (1995) 151:136–44. doi: 10.1164/ajrccm.151.1.7812543, PMID: 7812543

[32] Fagerås M Tomičić S Voor T Björkstén B Jenmalm MC. Slow salivary secretory iga maturation may relate to low microbial pressure and allergic symptoms in sensitized children. Pediatr Res. (2011) 70:572–7. doi: 10.1203/PDR.0b013e318232169e, PMID: 21857384

[33] Sandin A Björkstén B Böttcher MF Englund E Jenmalm MC Bråbäck L. High salivary secretory iga antibody levels are associated with less late-onset wheezing in ige-sensitized infants. Pediatr Allergy Immunol. (2011) 22:477–81. doi: 10.1111/j.1399-3038.2010.01106.x, PMID: 21332801

[34] Wu LC Zarrin AA. The production and regulation of ige by the immune system. Nat Rev Immunol. (2014) 14:247–59. doi: 10.1038/nri3632, PMID: 24625841

[35] Schwartz DP Buckley RH. Serum ige concentrations and skin reactivity to anti-ige antibody in iga-deficient patients. New Engl J Med. (1971) 284:513–7. doi: 10.1056/nejm197103112841002, PMID: 5100722

[36] Romero J Scadding G. Eosinophila in nasal secretions compared to skin prick test and nasal challenge test in the diagnosis of nasal allergy. Rhinology. (1992) 30:169–75. doi: 10.4193/Rhin10.4193/Rhin92.303, PMID: 1448673

[37] Halliwell B. Commentary for “Oxygen free radicals and iron in relation to biology and medicine: some problems and concepts. Arch Biochem Biophysics. (2022) 718:109151. doi: 10.1016/j.abb.2022.109151, PMID: 35181351

[38] Halliwell B. Oxidative stress and neurodegeneration: where are we now? J Neurochem. (2006) 97:1634–58. doi: 10.1111/j.1471-4159.2006.03907.x, PMID: 16805774

[39] Gaurav R Varasteh JT Weaver MR Jacobson SR Hernandez-Lagunas L Liu Q. The R213g polymorphism in sod3 protects against allergic airway inflammation. JCI Insight. (2017) 2:e95072. doi: 10.1172/jci.insight.95072, PMID: 28878123 PMC5621928

[40] Cuello-Garcia CA Fiocchi A Pawankar R Yepes-Nuñez JJ Morgano GP Zhang Y. World allergy organization-mcmaster university guidelines for allergic disease prevention (Glad-P): prebiotics. World Allergy Organ J. (2016) 9:10. doi: 10.1186/s40413-016-0102-7, PMID: 26962387 PMC4772464

[41] Patil AS Xu Y. Comprehensive metabolomics in mouse mast cell model of allergic rhinitis for profiling, modulation, semiquantitative analysis, and pathway analysis. Biomolecules. (2025) 15:109. doi: 10.3390/biom15010109, PMID: 39858503 PMC11763337

[42] Kang-Lee YA Harper AE. Effect of histidine intake and hepatic histidase activity on the metabolism of histidine *in vivo* . J Nutr. (1977) 107:1427–43. doi: 10.1093/jn/107.8.1427, PMID: 886385

[43] Wu X Zhu J Chen S Xu Y Hua C Lai L. Integrated metabolomics and transcriptomics analyses reveal histidine metabolism plays an important role in imiquimod-induced psoriasis-like skin inflammation. DNA Cell Biol. (2021) 40:1325–37. doi: 10.1089/dna.2021.0465, PMID: 34582699

[44] Maintz L Schwarzer V Bieber T van der Ven K Novak N. Effects of histamine and diamine oxidase activities on pregnancy: A critical review. Hum Reprod Update. (2008) 14:485–95. doi: 10.1093/humupd/dmn014, PMID: 18499706

[45] Yan B Lou H Wang Y Li Y Meng Y Qi S. Epithelium-derived cystatin sn enhances eosinophil activation and infiltration through il-5 in patients with chronic rhinosinusitis with nasal polyps. J Allergy Clin Immunol. (2019) 144:455–69. doi: 10.1016/j.jaci.2019.03.026, PMID: 30974106

[46] Miao P Jiang Y Jian Y Shi J Liu Y Piewngam P. Exacerbation of allergic rhinitis by the commensal bacterium streptococcus salivarius. Nat Microbiol. (2023) 8:218–30. doi: 10.1038/s41564-022-01301-x, PMID: 36635572 PMC10062442

