# Supplementary material for: Weizmannia coagulans BC99 regulates oxidative stress and serum metabolic pathways to improve allergic rhinitis: a randomized, double-blind, placebo-controlled trial
Source: Front Immunol. 2025 Oct 24;16:1654724. doi: 10.3389/fimmu.2025.1654724 (PMC12592054; doi:10.3389/fimmu.2025.1654724)
Supplement: Supplementary file 1 [file Table1.docx]

Supplementary Material

**Supplementary Table S1** Comparison of serum metabolites between the BC99 and placebo groups after 8-week intervention

| **Name** | **BC99** | **Placebo** | ***P* value** | **Fold-change** |
| --- | --- | --- | --- | --- |
| Pantothenol | 18.91899575 | 23.38665062 | 0.001511686 | 0.045196195 |
| N-Acetyl-L-phenylalanine | 15.4785629 | 19.10166926 | 3.62949E-05 | 0.081158929 |
| 2',6'-Dihydroxyacetophenone | 21.92044782 | 17.74981354 | 0.000367558 | 18.00885163 |
| O,O-Dimethyl malathion | 18.1108414 | 13.97676145 | 0.000172832 | 17.55828394 |
| 2-(2-Methylbenzamido)acetic acid | 15.75989157 | 18.63783191 | 3.62078E-06 | 0.136035931 |
| Cyclamic acid | 18.28454254 | 14.17036831 | 0.003852038 | 17.31768555 |
| Hexahydropyrrolo[1,2-a]pyrazine-1,4-dione | 11.73863553 | 14.93275933 | 0.000373084 | 0.109262951 |
| PS(18:2/0:0) | 18.62139611 | 21.7787763 | 9.02959E-05 | 0.11208148 |
| 5Z,9Z,21Z-hexacosatrienoic acid | 16.40770457 | 19.39497223 | 0.000186969 | 0.126108056 |
| SCHEMBL6037154 | 18.01278757 | 21.18431084 | 0.001300359 | 0.110988086 |
| Haloxon | 21.75375317 | 18.28516225 | 0.000484125 | 11.07005829 |
| 16beta,20S-dihydroxycholestan-3-one | 17.68336904 | 20.90357186 | 0.002828163 | 0.107305593 |
| Enterolactone 3''-glucuronide | 17.10987299 | 13.43801386 | 0.000614874 | 12.74499699 |
| Cromakalim | 15.12957701 | 18.07455808 | 0.000693681 | 0.129859092 |
| Benzoic acid, 5-amino-2-(sulfooxy)- | 17.75862562 | 13.56416454 | 0.003849442 | 18.30874599 |
| 4-Hydroxy-5-methyl-3(2H)-thiophenone | 20.06747511 | 22.94919047 | 0.001215909 | 0.135680438 |
| 6-Methoxy-3-(2-thiazolyl)-1H-indole | 13.99970357 | 16.3871612 | 3.68536E-05 | 0.191118901 |
| Ethionamide sulphoxide | 16.95442205 | 13.42393457 | 0.001126796 | 11.5553374 |
| Uracil mustard | 17.58329656 | 14.06596844 | 0.001537575 | 11.45041614 |
| Imidazole acetol-phosphate | 25.4142161 | 22.46281026 | 0.000415518 | 7.73502441 |
| Quercetagetin | 18.7373347 | 15.38547311 | 0.002018973 | 10.20965061 |
| Benzoyl chloride | 16.2691285 | 12.82061495 | 0.011293052 | 10.91706809 |
| (E)-3-methyl-6-(pent-3-en-1-yn-1-yl)-1,2-dithiine | 23.68290324 | 20.89524886 | 0.00044384 | 6.905062025 |
| p-Cresyl sulfate | 29.27306165 | 26.59000881 | 0.000343004 | 6.422134316 |
| 4(1H)-Pteridinone, 2-amino-7-(1,2-dihydroxypropyl)-5,6,7,8-tetrahydro- | 16.28388834 | 18.56884297 | 0.000299874 | 0.205191856 |
| Pyroglutamic acid | 22.27887188 | 25.62280736 | 0.014747477 | 0.09848614 |
| 7-Ketolithocholic acid | 18.01032706 | 20.68001429 | 0.006452907 | 0.15716074 |
| (E)-5-(3,4,5,6-Tetrahydro-3-pyridylidenemethyl)-2-furanmethanol | 16.45203853 | 19.286071 | 0.001498946 | 0.140239778 |
| 3,5-Dimethyl-1,2-cyclopentanedione | 23.25833464 | 20.55809586 | 0.001057987 | 6.499094728 |
| 2-Amino-4-methylpentanoic acid | 16.76814186 | 19.42275381 | 0.00090785 | 0.158811583 |
| (5Z,7E,24aE)-(1S,3R)-24a,24b-dihomo-9,10-seco-5,7,10(19),24a-cholestatetraene-1,3,25-triol | 17.56416287 | 19.93598414 | 0.003909169 | 0.193201572 |
| (2S,4S)-Monatin | 13.02118262 | 14.94374658 | 0.000896527 | 0.263785295 |
| Imazodan | 15.99774151 | 18.39472423 | 0.002691988 | 0.189861235 |
| Bicarbonate carbon | 18.12483306 | 15.82961061 | 0.000884712 | 4.908296652 |
| Tetrahydro-2-methyl-3-furanol | 17.93011746 | 20.60067345 | 0.015291531 | 0.15706613 |
| M8-Nelfinavir | 19.22978221 | 21.69871636 | 0.003927716 | 0.180624543 |
| Soyasapogenol D | 19.90645112 | 22.23953409 | 0.011809998 | 0.198459568 |
| Propyl 1-(propylsulfinyl)propyl disulfide | 21.59421547 | 20.25567014 | 1.73085E-09 | 2.528961949 |
| 2-Mercapto-3-furan-2-ylpropenoic acid | 17.00138452 | 19.48719867 | 0.008715854 | 0.178523494 |
| N-Eicosapentaenoyl Glutamine | 18.03083543 | 19.65756169 | 9.04365E-05 | 0.323822186 |
| lithocholic acid sulfate | 20.24564557 | 17.21110627 | 0.014361241 | 8.193837595 |
| 3R-aminononanoic acid | 16.8194542 | 14.21256108 | 0.017758818 | 6.091903635 |
| 5-Hexyltetrahydro-2-furanoctanoic acid | 19.2306244 | 21.46119145 | 0.014257617 | 0.213074957 |
| Citbrasine | 17.4958631 | 15.27141964 | 0.004609114 | 4.673305858 |
| 8-hydroxy-3E,5E,10E,12-tridecatetraen-2-one | 17.4446885 | 15.15562472 | 0.008296602 | 4.887388483 |
| Gentian Violet | 20.45583454 | 22.37831287 | 0.000911804 | 0.26380095 |
| tetracosahexaenoic acid | 14.93161089 | 17.02692016 | 0.002519548 | 0.23401789 |
| FT-0670561 | 15.71038216 | 13.20554135 | 0.025999129 | 5.675867081 |
| delta-Guanidinovaleric acid | 20.14131245 | 18.48710438 | 0.000940229 | 3.147503701 |
| Kuhlmanniquinol | 15.31001655 | 12.80784473 | 0.003654387 | 5.665376443 |
| Metoprolol acid | 18.20076178 | 20.29279825 | 0.00228073 | 0.23454937 |
| 5alpha-Cholestane-3beta,7alpha,12alpha,25,26-pentol | 17.03664447 | 19.12386607 | 0.002253808 | 0.235333466 |
| 12Z,16-heptadecadienoic acid | 17.82930943 | 19.62047614 | 0.001619694 | 0.288938286 |
| 2E,4E,6E,8E,10E,12E,14E,16E,18E,20E,22E-tetracosaundecaenal | 17.63420535 | 19.60228105 | 0.016592211 | 0.255593722 |
| Santowhite | 16.41316896 | 18.10685759 | 0.004634988 | 0.309135528 |
| Dinitrobenzene sulfonic acid | 16.48072936 | 13.94326267 | 0.008240653 | 5.805686561 |
| (R)-Rhazinilam | 13.18229276 | 14.87549379 | 0.002657211 | 0.309240026 |
| [[(1S,2S,5R)-5-Methyl-2-propan-2-ylcyclohexyl]-phenylphosphoryl]benzene | 19.75078636 | 21.52941722 | 0.001406406 | 0.291459864 |
| Glycolithocholic acid | 20.46436882 | 17.57737754 | 0.024693969 | 7.397261519 |
| Penicillanic acid | 15.36988856 | 13.06905339 | 0.045184691 | 4.927429293 |
| 1,1,1-Tri(butyryloxy)propane | 16.80507042 | 18.58519844 | 0.005089984 | 0.291157558 |
| Cbz-L-prolyl-D-proline | 16.84166226 | 18.69019598 | 0.007770949 | 0.277674439 |
| Solacetal B | 13.75211014 | 15.62852799 | 0.005185874 | 0.272359132 |
| 2-[4-(3-Hydroxypropyl)-2-methoxyphenoxy]-1,3-propanediol | 15.08644681 | 16.84675084 | 0.005238085 | 0.295185952 |
| 2,3,8,10-Tetrahydroxy[2]benzopyrano[4,3-b][1]benzopyran-7(5H)-one | 19.90933006 | 17.53473079 | 0.016557174 | 5.185917529 |
| DG(15:0/17:1/0:0)[iso2] | 16.64599289 | 18.32042136 | 0.011961378 | 0.313290196 |
| Deoxycholic acid | 23.16338099 | 20.94497572 | 0.021159593 | 4.653787289 |
| 1alpha,25-dihydroxy-24a,24b,24c-trihomo-22-thiavitamin D3 | 18.49783447 | 20.38004543 | 0.008592379 | 0.271267674 |
| Urobilin | 15.97143468 | 18.02631901 | 0.021292098 | 0.240667906 |
| 15S-HTPE | 18.24340842 | 16.20116407 | 0.010327078 | 4.118857871 |
| FT-0665222 | 18.58095569 | 16.17091459 | 0.016324067 | 5.314894659 |
| O-methoxycatechol-O-sulphate | 23.28394065 | 21.07246019 | 0.008425257 | 4.63150302 |
| 2-Amino-5-phosphonopentanoic acid | 16.4662568 | 14.18039883 | 0.025456009 | 4.876540279 |
| (-)-jasmonoyl-L-isoleucine | 14.08404059 | 16.0067175 | 0.031325638 | 0.263764644 |
| Benzolamide | 18.11516458 | 16.18722927 | 0.01676963 | 3.805102495 |
| MG(LTE4/0:0/0:0) | 22.4602534 | 20.18425666 | 0.025611181 | 4.843321377 |
| 28:3(5Z,9Z,21Z) | 18.28841007 | 19.76952443 | 0.002762008 | 0.358212015 |
| Arabinosylhypoxanthine | 21.9184082 | 21.00924174 | 3.35054E-09 | 1.877960159 |
| gamma- 12(13)-EpODE | 17.72016301 | 19.25526936 | 0.011035296 | 0.3450539 |
| Propylparaben sulfate | 16.94491374 | 14.55963232 | 0.041550634 | 5.224458181 |
| 5-Methoxy-galloyl-1,4-galactarolactone | 17.76425167 | 16.08145774 | 0.01357887 | 3.210490954 |
| Landiolol | 16.53231181 | 18.19507061 | 0.004093716 | 0.315834615 |
| 2-(p-Acetamidophenyl)-2-ethylglutarimide | 17.34473969 | 19.02413819 | 0.012370274 | 0.312212782 |
| Clocapramine | 14.9901199 | 16.71570297 | 0.005669613 | 0.30237629 |
| Cocaine-alcohol | 16.31199128 | 14.52836301 | 0.00866954 | 3.442909532 |
| N-(2-Hydroxyisobutyl)-2,4,8,10,12-tetradecapentaenamide | 14.30381884 | 15.87598965 | 0.009705185 | 0.336301984 |
| 3-Hydroxymonoethylglycinexylidide | 15.20713136 | 13.23857566 | 0.027563797 | 3.913761105 |
| 2-Ethylbenzothiazoline sulfonic acid | 24.09829453 | 22.73192281 | 0.001040167 | 2.578213469 |
| 3-(Acetyloxy)-2-hydroxypropyl icosanoate | 15.45719815 | 13.49290408 | 0.04885636 | 3.902217164 |
| Homomethionine | 18.48355535 | 17.03265665 | 0.001542609 | 2.733782928 |
| bendazac | 18.97377592 | 20.11379114 | 0.000863815 | 0.453754791 |
| [2-methoxy-4-(3,5,7-trihydroxy-4-oxo-2,3-dihydrochromen-2-yl)phenyl] hydrogen sulate | 16.26954579 | 14.5857166 | 0.0282302 | 3.212795596 |
| 4-Hydroxy-2-oxobutanoic acid | 19.71520788 | 21.86398072 | 0.021285 | 0.22550435 |
| Cannabidivarin | 19.24633904 | 20.50041593 | 0.000537479 | 0.419261746 |
| 7-Chloro-6-demethylcepharadione B | 17.29699152 | 15.60825254 | 0.016689998 | 3.223748021 |
| CHEMBL30930 | 17.20824969 | 18.64398388 | 0.012887406 | 0.369658709 |
| 15:6(2Z,4E,6Z,8E,12E,14)(6Me,8Me,10Me[S],13Me) | 18.6228779 | 19.6848308 | 0.000266286 | 0.478983247 |
| Brinzolamide | 20.78962913 | 19.36696693 | 0.003540411 | 2.68079742 |
| 5-Hydroxyoxindole | 21.3610211 | 20.08175751 | 0.001224103 | 2.427150524 |
| TRICIRIBINE PHOSPHATE | 20.74188006 | 19.45006027 | 0.002401755 | 2.448366945 |
| N-Acetyl-L-glutamyl 5-phosphate | 18.19352019 | 16.54761315 | 0.009227837 | 3.129445485 |
| Methyl 9-hydroxynonanoate | 17.82450319 | 18.96955961 | 0.001662126 | 0.452172005 |
| 1,3-Propane sultone | 19.1067858 | 20.57634957 | 0.029283276 | 0.361091466 |
| Phenylalanylmethionine | 22.19084318 | 20.97723629 | 0.001958843 | 2.319167284 |
| 2-heptenal | 16.64720946 | 18.34262793 | 0.031285727 | 0.308765085 |
| 3alpha-Hydroxy-5beta-chol-14-en-24-oic Acid | 23.23973667 | 21.34598996 | 0.045944926 | 3.715990229 |
| 5alpha-Cholestane-3alpha,7alpha,26-triol | 18.36241197 | 19.35633061 | 0.00031154 | 0.502112087 |
| Lupinic acid | 14.86861334 | 13.1396498 | 0.03885712 | 3.31489584 |
| Cholylaspartic acid | 16.43558809 | 17.85209509 | 0.012221911 | 0.374618227 |
| 4-Hydroxy Duloxetine | 18.75324439 | 17.42499831 | 0.007946448 | 2.510972247 |
| Cadabicilone | 15.83267197 | 17.30546697 | 0.015961411 | 0.360283628 |
| Gcdcs | 15.89933871 | 17.34509817 | 0.01530341 | 0.36709886 |
| Annuolide G | 16.81544586 | 18.09447811 | 0.018651048 | 0.41207183 |
| N-(3beta,7beta,dihydroxycholest-5-en-24-oyl) glycine | 19.65677469 | 17.6060128 | 0.048520275 | 4.143247167 |
| 9'-Carboxy-gamma-chromanol | 18.3209577 | 19.30289363 | 0.002380759 | 0.506299887 |
| Monoacetyldiglyceride | 19.66496834 | 18.42201395 | 0.00642567 | 2.366827219 |
| Heteranthin | 16.85655676 | 18.2306075 | 0.008678403 | 0.385806476 |
| Dimethylaminocinnamaldehyde | 17.90495837 | 16.08780986 | 0.035497486 | 3.52384023 |
| 4,4'-Dipyridyl disulfide | 24.01648601 | 23.31535576 | 3.35389E-07 | 1.625777982 |
| 5beta-Cholestane-3alpha,7alpha,12alpha,22-tetrol | 19.39949877 | 20.78129833 | 0.042602766 | 0.383739833 |
| HMBOA hexose | 22.42336899 | 23.43761834 | 0.001293733 | 0.495085862 |
| 3-[4-(sulfooxy)phenyl]propanoic acid | 17.3667106 | 18.6512415 | 0.016128245 | 0.41050426 |
| PS(20:5/0:0) | 17.8271228 | 18.82495937 | 0.001306102 | 0.500750348 |
| 4-Hprog | 17.45607059 | 19.00760686 | 0.022114244 | 0.341146596 |
| SCHEMBL7292028 | 18.9498232 | 20.37359515 | 0.042019381 | 0.37273651 |
| (Z)-Resveratrol 3-(2''-sulfoglucoside) | 16.03823136 | 17.25741598 | 0.031291245 | 0.42952541 |
| Oxaprozin glucuronide | 14.93227713 | 16.45254725 | 0.040136448 | 0.348620639 |
| estradiol cypionate | 22.23430308 | 23.42975574 | 0.005918919 | 0.436649426 |
| Arctinone A | 21.29835884 | 20.05619938 | 0.008139917 | 2.365523438 |
| (3-Aminopropyl)(n-butyl)phosphinic acid | 16.53758966 | 17.4295516 | 0.000414866 | 0.538880791 |
| 2-(Pentylamino)ethanol | 16.86284625 | 15.56077839 | 0.011876139 | 2.465820637 |
| Deoxycholic acid 3-glucuronide | 15.10143647 | 13.54031348 | 0.033785629 | 2.950834463 |
| Velagliflozin | 15.00783507 | 16.18965392 | 0.010829106 | 0.440795423 |
| Eicosen-1-ol | 18.2457485 | 19.41539677 | 0.003746405 | 0.444529707 |
| FT-0665875 | 14.89053804 | 13.49263608 | 0.041334492 | 2.635180834 |
| Dihyroxy-1H-indole glucuronide I | 17.21489458 | 15.63845775 | 0.038980184 | 2.982323644 |
| Threonylglutamine | 18.70159138 | 19.86818696 | 0.006658565 | 0.44547131 |
| Carboxymethyl chitosan | 16.83984127 | 18.16354126 | 0.022461604 | 0.399509026 |
| Phenylacetylglutamine | 23.24823772 | 22.12337232 | 0.005371703 | 2.180811983 |
| 2-Linoleoyl Glycerol | 17.25193104 | 18.51287269 | 0.025555387 | 0.417271516 |
| Terretonin | 17.60429997 | 18.82350186 | 0.010176966 | 0.429520265 |
| 2-Phenylacetamide | 19.726549 | 18.50732306 | 0.009294279 | 2.328217665 |
| Methyl N-methylanthranilate | 16.53138128 | 17.62878655 | 0.011490141 | 0.467356295 |
| D-Methionine (S)-S-oxide | 21.41316148 | 20.60529998 | 3.28357E-05 | 1.750614588 |
| 9,13-dihydroxy-10-ethoxy-11-octadecenoic acid | 17.25189799 | 18.40742703 | 0.01016105 | 0.448901544 |
| Indoxyl sulfate | 26.83915472 | 25.72075876 | 0.00476094 | 2.171054525 |
| 3-oxohexacosanoic acid | 18.65636261 | 19.63423603 | 0.001975758 | 0.507727596 |
| Tenoxicam | 20.14666132 | 19.01643976 | 0.006926634 | 2.188923537 |
| Dansylamide | 17.35708405 | 18.310032 | 0.005083316 | 0.516575831 |
| Purpald | 24.18513251 | 24.99533461 | 0.001461801 | 0.57030196 |
| Boviquinone 4 | 18.41605547 | 19.51864238 | 0.006971495 | 0.46568073 |
| Glycochenodeoxycholic acid | 25.2985513 | 26.37174919 | 0.006603909 | 0.475264355 |
| 2-O-alpha-D-Glucopyranosyl-O-beta-D-galactopyranosylhydroxylysine | 15.34073322 | 16.6675785 | 0.040430035 | 0.398638987 |
| 5'-Carboxy-gamma-chromanol | 18.15197021 | 19.14460838 | 0.011239268 | 0.502557934 |
| Prehumulinic acid | 19.72696462 | 18.45281311 | 0.024073768 | 2.418565326 |
| Cohulupone | 18.24406707 | 19.11528838 | 0.001072428 | 0.54668386 |
| N-trans-p-Coumaroyloctopamine | 16.39534592 | 17.51713717 | 0.034357777 | 0.45952293 |
| Iridodial glucoside tetraacetate | 18.70116219 | 19.69959059 | 0.02282153 | 0.500544972 |
| (E)-2-O-Cinnamoyl-beta-D-glucopyranose | 16.02093585 | 17.10276113 | 0.015776778 | 0.472430732 |
| 6-Carboxy-5,6,7,8-tetrahydropterin | 22.81367644 | 21.71923159 | 0.007129571 | 2.135308983 |
| 2,5-Dihydroxybenzoic acid | 16.41536358 | 17.44262981 | 0.009488643 | 0.490638983 |
| oscr#35 | 19.91231563 | 21.10757896 | 0.021862267 | 0.436706731 |
| Methyl-(10R)-hydroxy-(11S,12S)-epoxy-(5Z,8Z,14Z)-eicosatrienoate | 18.69661322 | 19.58660644 | 0.00572078 | 0.539616655 |
| Castillene D | 21.86834964 | 21.32619643 | 5.34277E-10 | 1.456144182 |
| DG(12:0/20:1/0:0)[iso2] | 18.80762394 | 19.8210626 | 0.02653856 | 0.495364138 |
| Cucurbitacin IIa | 17.00764564 | 18.05410593 | 0.009909332 | 0.4841546 |
| Uridine 2'-phosphate | 16.50865152 | 17.62731914 | 0.019643973 | 0.460518935 |
| Glucosaminylmuramyl-2-alanine-D-isoglutamine | 19.70262024 | 20.79708606 | 0.00935606 | 0.468309489 |
| (6beta,8betaOH)-6,8-Dihydroxy-7(11)-eremophilen-12,8-olide | 17.81717712 | 18.93223124 | 0.021630025 | 0.461673837 |
| Dansyl chloride | 21.05294587 | 20.00846502 | 0.007804058 | 2.062623982 |
| 2-methyl-tridecanedioic acid | 17.96902298 | 16.93944088 | 0.018013704 | 2.041432838 |
| 11-acetoxy-3beta,6alpha-dihydroxy-9,11-seco-5alpha-cholest-7-en-9-one. | 18.45569552 | 19.41279143 | 0.030844904 | 0.515092732 |
| N-Choloylglycine | 22.31187607 | 23.39176199 | 0.01244552 | 0.473066228 |
| MG(18:0/0:0/0:0) | 17.51220045 | 18.50495039 | 0.039218908 | 0.502519006 |
| cis-Parinaric acid | 19.62559191 | 20.37870743 | 0.001329807 | 0.593320891 |
| 1-Methoxy-2-propanol | 16.80747964 | 17.68379235 | 0.009426916 | 0.544757968 |
| Dimethyl 3-methoxy-4-oxo-5-(8,11,14-pentadecatrienyl)-2-hexenedioate | 17.5067187 | 18.45456197 | 0.006714444 | 0.518406867 |
| 1-{2-[(3-Ethylphenyl)amino]-2-oxoethyl}-6-oxo-1,6-dihydropyridine-3-carboxylic acid | 17.13138257 | 17.9161591 | 0.003308716 | 0.580441862 |
| ibho#24 | 19.96260582 | 21.03288311 | 0.018146721 | 0.476227459 |
| PA(0:0/18:1) | 17.12558752 | 18.18777413 | 0.017828552 | 0.478905659 |
| O-Ureidohomoserine | 21.69879472 | 21.00959221 | 0.000930299 | 1.61239198 |
| Cinnamoyl-homoserine lactone | 17.67479184 | 16.65100099 | 0.015468818 | 2.03325457 |
| Pentyl acetate | 19.86450875 | 19.1202706 | 0.001427223 | 1.675089461 |
| Armillane | 17.09811719 | 17.99327661 | 0.034839554 | 0.537687777 |
| Bufalin sulfate | 20.68742021 | 21.52534126 | 0.016516004 | 0.559449165 |
| DG(16:0/i-16:0/0:0) | 18.13988708 | 19.02523169 | 0.035282834 | 0.541358196 |
| Phosphonate analog | 18.13850776 | 18.92096144 | 0.000456871 | 0.581377169 |
| Hippuric acid | 22.342396 | 21.32813231 | 0.014683012 | 2.019871738 |
| Iberin-N-acetyl-cysteine | 19.6683642 | 18.6838555 | 0.032068361 | 1.978639381 |
| Piperonyl sulfoxide | 22.8671998 | 23.62461908 | 0.038865512 | 0.591553568 |
| Sulfoacetic acid | 22.34499939 | 21.8634868 | 3.87688E-08 | 1.396206743 |
| Lewis b | 18.32816644 | 19.33356562 | 0.022917569 | 0.498132284 |
| omega-hydroxy enanthoic acid | 19.46327545 | 20.23013676 | 0.003388982 | 0.587694658 |
| 2-(4-Hydroxy-3,5-di-tert-butylphenylthio)-hexanoic acid | 18.18026725 | 19.0127576 | 0.017382457 | 0.561559053 |
| Cavipetin D | 17.63337916 | 18.41292856 | 0.005857219 | 0.582548714 |
| 17S-HpDHA | 18.2012914 | 18.87999159 | 0.006843926 | 0.624727873 |
| Phosphatidylinositol 4,5-diphosphate | 16.63865123 | 17.58424199 | 0.046644255 | 0.519216898 |
| Glutathione bicarbonate | 17.19341089 | 17.84752508 | 0.002583252 | 0.635465544 |
| ibho#22 | 21.29286184 | 22.04673204 | 0.009340982 | 0.593010602 |
| Umbelliferone sulfate | 23.40791306 | 22.52952122 | 0.032257816 | 1.838324994 |
| 14-HpEPE | 19.28138985 | 19.97979078 | 0.005688702 | 0.616254878 |
| Pentadeca-5,8,11-trienedioylcarnitine | 17.79671284 | 16.79424594 | 0.03045815 | 2.003422771 |
| plaunotol | 21.93517836 | 22.71558774 | 0.022037253 | 0.582201566 |
| PC(18:3/0:0) | 23.32676782 | 23.98084466 | 0.001681552 | 0.635481993 |
| Decarbamoylsaxitoxin | 23.96794971 | 24.63125403 | 0.006362543 | 0.631430421 |
| (22E,24R)-Stigmasta-4,22-diene-3,6-dione | 18.73977462 | 19.3791238 | 0.007564667 | 0.642002503 |
| 3alpha,7alpha,11alpha-Trihydroxy-12-oxo-5beta-cholan-24-oic Acid | 17.13357368 | 17.82630165 | 0.017545982 | 0.618682887 |
| Prenyl cis-caffeate | 17.24572897 | 16.34652746 | 0.013845275 | 1.865033446 |
| Leontogenin | 17.58021004 | 18.44888498 | 0.033586759 | 0.547649612 |
| 6-((Z)-14-hydroxypentadec-8-en-1-yl)salicylic acid | 19.13422521 | 19.78830067 | 0.009191444 | 0.635482609 |
| 3-(3-Oxobutanoyloxy)butanoic acid | 16.64089048 | 17.35833608 | 0.014419818 | 0.608173303 |
| (+)-Serradiol | 20.89632674 | 21.51901849 | 0.0048841 | 0.64945805 |
| 2-Hexenoylcholine | 18.68114991 | 17.81136735 | 0.024528101 | 1.827387458 |
| Ophiobolin Q | 18.24024688 | 18.9014237 | 0.00594313 | 0.632362265 |
| 4-Methyl-3-heptyl linoleate | 18.54061116 | 19.33814838 | 0.022112993 | 0.575330469 |
| Diethylpropion | 21.34595814 | 21.87799515 | 0.00034838 | 0.691577574 |
| 3-Methyl-3-butenyl hexadecanoate | 18.34416221 | 19.07204914 | 0.027093599 | 0.603787615 |
| 3alpha,23-O-isopropylidenyl-3alpha,23-dihydroxylup-20(29)-en-28-oic acid | 18.09137793 | 18.96034462 | 0.036579099 | 0.547538876 |
| PC(18:1/2:0) | 19.49797058 | 20.0003512 | 0.000196756 | 0.705940934 |
| methyl-6S-hydroxy-2E,4E-decadienoate | 16.45370684 | 17.21586057 | 0.042048001 | 0.589615466 |
| DG(8:0/0:0/15:0) | 18.33825548 | 19.04733325 | 0.016155027 | 0.611711044 |
| 1,3-Benzodioxole | 21.72116468 | 20.88863798 | 0.021343542 | 1.780801479 |
| HOMATROPINE | 17.40517462 | 18.07702002 | 0.002670108 | 0.627703256 |
| Anandamide (20:2, n-6) | 20.3348792 | 20.91163724 | 0.003306735 | 0.670468732 |
| PE(0:0/18:3) | 20.74982722 | 21.35867643 | 0.006077457 | 0.655719541 |
| 5alpha-Cholanoic acid | 19.16043494 | 19.8491353 | 0.016092324 | 0.620412493 |
| Feruloylcholine | 20.45376024 | 19.64697293 | 0.031067594 | 1.749311616 |
| (6R,7S)-6,7-Epoxy-1,3-tetradecadiyne | 18.45633756 | 19.09993939 | 0.018995122 | 0.640112846 |
| 5-Hexadecanoylaminofluorescein | 20.43093726 | 21.04093931 | 0.001891163 | 0.655195772 |
| 6-Methyl-5-hepten-2-one propyleneglycol acetal | 17.32212528 | 16.49486314 | 0.041859345 | 1.774314971 |
| Acetyl-D-carnitine | 22.42502682 | 21.773799 | 0.018597896 | 1.570504221 |
| 2-(2-Cyclohexylethylhydrazono)propionic acid | 16.06852258 | 16.70254827 | 0.023669541 | 0.644375838 |
| alpha,beta-Methylene ATP | 19.95121892 | 20.67469279 | 0.025648026 | 0.60563737 |
| Phenol sulphate | 27.75889612 | 27.02572652 | 0.047838839 | 1.662287124 |
| Rivafurazon | 18.05469123 | 18.77248423 | 0.034748207 | 0.608026879 |
| Glyceryl lactopalmitate | 16.66025093 | 17.31618418 | 0.016786173 | 0.634664809 |
| N-oleoyl alanine | 18.74012818 | 19.08260723 | 4.07948E-06 | 0.788684914 |
| 3-Amino-2-hydroxy-4-phenylbutanoic acid | 23.62495465 | 23.94015079 | 1.26401E-10 | 0.80374171 |
| (22alpha)-hydroxy-campesterol | 19.00409002 | 19.64817487 | 0.016370335 | 0.639898572 |
| N-3-hydroxy-13-methyl-hexadecanoyl glycyl-L-serine | 21.35437027 | 21.85493393 | 0.000919939 | 0.70683057 |
| octadec-11Z-enol | 21.45343154 | 20.72852224 | 0.040006762 | 1.652796726 |
| 9-B1-PhytoP | 17.18474862 | 16.38556857 | 0.03811054 | 1.740111856 |
| Propionic acid, 2-methyl-2-((piperidinomethyl)thio)- | 18.86597385 | 19.38470082 | 0.001021925 | 0.697987464 |
| 4-Amino-1,8-naphthalimide | 16.95870026 | 17.5311939 | 0.008220268 | 0.672453475 |
| Annocherin A | 19.16477711 | 19.70484725 | 0.006875805 | 0.687737472 |
| (5Z,7E)-(1S,3S)-3-methyl-9,10-seco-5,7,10(19)-cholestatriene-1,25-diol | 19.76173071 | 20.39322547 | 0.039892568 | 0.645507269 |
| Muricinine | 17.15826217 | 17.76415708 | 0.01167992 | 0.657063675 |
| Bakkenolide C | 18.88664375 | 19.49651759 | 0.023628773 | 0.655253999 |
| Anthenoside A1 | 19.48803568 | 19.02208459 | 0.000847416 | 1.381227615 |
| 7,7-dimethyl-5Z,8Z-eicosadienoic acid | 17.10828317 | 17.65374824 | 0.014858622 | 0.685170494 |
| 22:5(4Z,7Z,10Z,13Z,16Z) | 22.83258204 | 22.17725409 | 0.029176245 | 1.574973932 |
| Myrsinone | 18.46985771 | 19.05726821 | 0.03178534 | 0.665536409 |
| Propan-2-yl 2-hydroxy-4-methylsulfanylbutanoate | 17.5416649 | 16.85682025 | 0.01490916 | 1.607528874 |
| Olsalazine | 18.53936506 | 18.9951165 | 9.04295E-05 | 0.729130299 |
| Vitamin K1 | 17.88519683 | 18.44133173 | 0.013247651 | 0.68012183 |
| Fosamprenavir | 19.19665631 | 19.79109037 | 0.016035613 | 0.662304212 |
| Sterculynic acid | 19.63021065 | 19.12048402 | 0.009644258 | 1.423780384 |
| 1-(2-methoxy-5Z,19Z-hexacosadienyl)-sn-glycero-3-phosphoethanolamine | 16.32692123 | 16.93453199 | 0.024792781 | 0.656282667 |
| 6-[5]-ladderane-hexanoic acid | 18.99965321 | 19.61043186 | 0.011483232 | 0.654843174 |
| PE(20:5/0:0) | 20.87724663 | 21.37960546 | 0.002317913 | 0.705951592 |
| Rhubafuran | 18.14328893 | 18.60007839 | 0.000871884 | 0.728605875 |
| ent-4-epi-11-F4t-NeuroP | 18.86182117 | 19.2896898 | 0.00254281 | 0.74335918 |
| Promestrienum | 19.42240634 | 19.97310263 | 0.018300282 | 0.682690561 |
| PE(P-18:0/20:4) | 20.76402042 | 21.32859393 | 0.032376923 | 0.676155272 |
| Colupone | 18.75537204 | 19.29363109 | 0.011978245 | 0.688601368 |
| 5-(L-alanin-3-yl)-2-hydroxy-cis,cis-muconate 6-semialdehyde | 17.74769268 | 18.33906009 | 0.016294313 | 0.663713531 |
| 1,3-Dibenzylisoquinoline | 20.84596518 | 20.47210255 | 3.40668E-05 | 1.295817578 |
| 6-Methyl-N-tetrazol-5-yl-2-pyridinecarboxamide | 19.48455581 | 19.98335289 | 0.003566947 | 0.707696612 |
| 10,14-octadecadiynoic acid | 22.73978712 | 22.1952387 | 0.023275471 | 1.458563729 |
| 24-vinyloxy-cholest-5,23Z-dien-3beta-ol | 19.68243868 | 20.20205267 | 0.029152072 | 0.697558448 |
| 9(10)-EpOME | 19.46458661 | 20.11491492 | 0.047864016 | 0.637135308 |
| Threonylcysteine | 19.5950626 | 20.06733576 | 0.001653165 | 0.720827942 |
| Ethanol, 2-(pentyl(5-(1-piperidinyl)(1,2,4)triazolo(1,5-a)pyrimidin-7-yl)amino)- | 17.18310541 | 17.81798079 | 0.015361161 | 0.643996439 |
| Diallyl sulfone | 19.47514887 | 18.84918161 | 0.015407802 | 1.543245154 |
| N-(2-Hydroxyethyl)ethylenediaminetriacetic acid | 17.45716178 | 18.16824519 | 0.035558773 | 0.610861231 |
| tetradecyl propionate | 20.19842589 | 19.75811998 | 0.010326575 | 1.356892012 |
| Tuberoside | 18.25107873 | 18.84651575 | 0.04775719 | 0.661843941 |
| a,b-dihydroxy-isobutyric acid | 21.7838839 | 21.3824804 | 0.001960688 | 1.320792198 |
| 3-(Methylthio)propanal | 17.93332841 | 17.41657427 | 0.029273507 | 1.430732682 |
| 5S-HpEPE | 21.13993507 | 21.66932712 | 0.031532714 | 0.692846642 |
| 3-oxo-docosanoic acid | 18.36933193 | 18.74902217 | 0.000416636 | 0.768602598 |
| TRIETHYLENE GLYCOL | 23.57823195 | 23.05093716 | 0.012995499 | 1.441224212 |
| 1beta-butyl-1alpha,25-dihydroxyvitamin D3 | 20.20874861 | 20.75182254 | 0.043712873 | 0.686307044 |
| 13-Demethyl tacrolimus | 22.15770355 | 21.82082348 | 0.00011764 | 1.263022268 |
| Paramethadione | 18.37801697 | 18.94694483 | 0.029323683 | 0.67411757 |
| cis-Linoleic acid | 18.50920228 | 18.05180639 | 0.00916944 | 1.373061155 |
| 12-[Methyl-(4-nitro-2,1,3-benzoxadiazol-7-yl)amino]octadecanoic acid | 21.48326471 | 21.11689593 | 0.000220167 | 1.289104099 |
| 18,25-dihydroxyvitamin D3 | 20.4333864 | 20.91735667 | 0.043290888 | 0.715007228 |
| Laccarin | 20.72565869 | 21.21918877 | 0.014932452 | 0.710285001 |
| Thiocyanic acid, p-aminophenyl ester | 18.39652096 | 17.83853415 | 0.037800868 | 1.472213402 |
| Dehydroacetic acid | 18.790317 | 19.31678783 | 0.03336728 | 0.694250959 |
| 17Z-Docosenoic acid | 21.6192765 | 22.04299771 | 0.018786916 | 0.745499242 |
| Cerotic acid(d3) | 23.8510039 | 24.2683154 | 0.012306645 | 0.748818766 |
| PE(20:1/0:0) | 19.07604323 | 19.70034498 | 0.034453226 | 0.648733682 |
| FAHFA(14:11/3-O-10:0) | 18.31744035 | 17.75959773 | 0.029210907 | 1.472066279 |
| (all-E)-1,7-bis(4-hydroxyphenyl)-1,4,6-heptatrien-3-one | 25.03212136 | 25.39477369 | 0.00175895 | 0.777733432 |
| Elaidamide | 19.07462761 | 19.36008542 | 1.92024E-05 | 0.820481205 |
| trans-brassidic acid | 22.42831336 | 22.89438798 | 0.037523275 | 0.723931643 |
| Amidinophenylpyruvic acid | 20.67673866 | 21.08903939 | 0.006587017 | 0.751424085 |
| Isohumbertiol | 18.11671152 | 18.57511143 | 0.015099816 | 0.727793002 |
| PC(O-18:1/2:0) | 20.5772314 | 21.17341207 | 0.035845768 | 0.661502874 |
| (Z)-5-(heptadec-8-en-1-yl)resorcinol | 19.06405493 | 19.56706165 | 0.041217028 | 0.705634633 |
| arabinofuranosylguanine | 20.60398054 | 20.15679236 | 0.00426966 | 1.363380432 |
| PE(20:2/0:0) | 19.19694379 | 19.65955811 | 0.023089001 | 0.725670074 |
| Mono-(2-ethylhexyl) phthalate | 19.35012585 | 19.69366979 | 0.001630718 | 0.788102978 |
| Retinol acetate | 19.8498928 | 19.44615674 | 0.019922142 | 1.32292939 |
| methyl 5S,6R-epoxy-7-eicosynoate | 19.34849039 | 19.79468329 | 0.006456204 | 0.733977169 |
| Lupulone | 19.20874421 | 19.68615295 | 0.035994522 | 0.71826656 |
| 2-Undecyl-thiazolidine-4-carboxylic acid | 20.31761455 | 20.74898163 | 0.006123718 | 0.741558762 |
| 1-Methoxypyrene | 19.04425883 | 19.42826627 | 0.002410933 | 0.766306028 |
| N-(hexadecanoyl)-homoserine lactone | 17.96520084 | 17.55923913 | 0.019369182 | 1.32497185 |
| C25:3 6,7-Epoxy highly branched isoprenoid | 17.71368163 | 18.28737977 | 0.047185838 | 0.671892278 |
| (1R,2R)-3-oxo-2-pentyl-cyclopentanehexanoic acid | 19.03261117 | 19.48173036 | 0.012842406 | 0.732489916 |
| (E)-2-Hexenyl (E)-7,9-decadienoate | 20.91582131 | 20.51101731 | 0.015805468 | 1.323909031 |
| Pritelivir | 20.83477736 | 20.55133145 | 0.000125216 | 1.217098487 |
| 24-methylencycloartan-12-oxo-3beta,22R-diol | 19.32879192 | 19.77640744 | 0.046361849 | 0.733253766 |
| Phosphite | 21.4836979 | 21.91165011 | 0.035772928 | 0.743316113 |
| N-Acetyl-L-tyrosine | 16.82856384 | 17.26572992 | 0.010218708 | 0.738584006 |
| Albifylline | 18.27900852 | 18.7836698 | 0.011602579 | 0.704825841 |
| AKOS022198169 | 17.794067 | 18.26327301 | 0.029173888 | 0.722362042 |
| 5-Hydroxy-1-methylpyrrolidin-2-one | 21.41170264 | 21.84032461 | 0.006916667 | 0.742971119 |
| Paromomycin I | 21.31138676 | 21.72034983 | 0.01474099 | 0.753164516 |
| pyrrolobenzodiazepine | 20.25601199 | 19.86799875 | 0.011064971 | 1.308590078 |
| 2-Naphthyl dihydrogen phosphate | 18.14407739 | 18.533635 | 0.015682479 | 0.76336365 |
| Adrenic Acid | 23.29820601 | 22.8301759 | 0.040535756 | 1.383219498 |
| 13-Docosenamide | 29.79139144 | 30.22537079 | 0.043905142 | 0.740217244 |
| 5R-HpEPE | 17.69516233 | 18.05234195 | 0.019812824 | 0.78068929 |
| Chaetoglobosin N | 25.06973626 | 25.54703291 | 0.038496144 | 0.718322373 |
| Rosoxacin | 21.45645028 | 21.94424185 | 0.042723688 | 0.71311588 |
| 2-(Dimethylamino)octadec-4-ene-1,3-diol | 17.58990387 | 17.96476527 | 0.034374238 | 0.771179495 |
| (2R,3S)-3-(6-Amino-9H-purin-9-yl)nonan-2-ol | 19.62126949 | 20.03568926 | 0.029217904 | 0.750321204 |
| N-Ethyl-N'-nitro-N-nitrosoguanidine | 21.6822523 | 21.96298172 | 0.000274736 | 0.823174719 |
| (S)-2-Acetamido-3-(4-chlorophenyl)propanoic acid | 17.95561097 | 18.26569687 | 0.000127364 | 0.806593736 |
| N-Acetyl-S-(N-methylcarbamoyl)cysteine | 21.29989515 | 21.75134158 | 0.019043581 | 0.731309276 |
| L-Nicotianine | 20.56282262 | 20.93859167 | 0.028230294 | 0.770694474 |
| (±)-Pelletierine | 19.38690966 | 18.90761669 | 0.027857057 | 1.394060302 |
| PC(20:1/0:0) | 23.33071067 | 23.88101955 | 0.035749275 | 0.68287391 |
| N-Acetylornithine | 21.31055637 | 21.78070714 | 0.021933152 | 0.721889151 |
| Threonylglycine | 24.87116483 | 25.18356973 | 0.000888975 | 0.805298243 |
| Spongouridine | 19.16269452 | 18.80471283 | 0.010828596 | 1.281631664 |
| 5-Hydroxyisourate | 22.14801416 | 22.63079144 | 0.027291862 | 0.715598722 |
| 22:2(5Z,9Z)(13Me,17Me,21Me) | 19.9811926 | 20.43195311 | 0.049691414 | 0.731657058 |
| Lipstatin | 21.89871127 | 22.36886231 | 0.027802691 | 0.721889016 |
| gamma-Cadinol | 18.75878334 | 19.16267348 | 0.04115289 | 0.755817521 |
| D-Phenylalanyl-L-2-piperidinecarbonyl-N-(4-nitro phenyl)-L-argininamide | 19.98091854 | 19.72041879 | 0.000377593 | 1.197893586 |
| Pentrinitrol | 18.76634698 | 19.19661851 | 0.045027677 | 0.742122099 |
| 5-Nitro-2-(3-phenylpropylamino)benzoic acid | 23.80043688 | 24.2238401 | 0.036405762 | 0.745663577 |
| 4-Hydroxyretinoic acid glucuronide | 22.16763463 | 21.92832982 | 5.83886E-05 | 1.180423711 |
| Levoglucosan | 18.93344528 | 18.5473663 | 0.006617873 | 1.306836797 |
| PE(18:2/0:0) | 26.42048054 | 26.788972 | 0.013154506 | 0.774592018 |
| Hopane-29-acetate | 20.8944595 | 21.35667291 | 0.048817246 | 0.725871756 |
| PA(15:0/0:0) | 22.85363456 | 22.51936918 | 0.030127478 | 1.260735279 |
| Zooxanthellactone | 23.39444974 | 23.06190628 | 0.015587391 | 1.259231433 |
| 16-Glutaryloxy-1alpha,25-dihydroxyvitamin D3 | 21.09894079 | 21.44639031 | 0.01389518 | 0.78597236 |
| 6-Aminopurin-8-one | 19.34320567 | 19.03710591 | 0.002144948 | 1.236360752 |
| 1-O-(2-methoxy-hexadecyl)-sn-glycerol | 18.42113512 | 18.74818131 | 0.007551202 | 0.797166951 |
| 3-Methyl-8-(2-methylpropyl)-7H-purine-2,6-dione | 17.6485953 | 18.00676004 | 0.004070896 | 0.780156389 |
| Dimethyl mercaptosuccinate | 19.24884046 | 18.8604195 | 0.031936662 | 1.30895995 |
| Blebbistatin | 20.01212575 | 19.66741787 | 0.017340254 | 1.269893822 |
| (2S,3R,4E,8E)-3-Hydroxy-2-[methyl(stearoyl)amino]-4,8-octadecadien-1-yl hydrogen sulfate | 21.66119578 | 22.03616961 | 0.017175682 | 0.771119404 |
| 3h-Serotonin | 19.59827692 | 19.20284011 | 0.039633566 | 1.315340951 |
| Citflavanone | 20.31159322 | 19.98566765 | 0.016039631 | 1.253468349 |
| 8-Hydroxyapigenin 8-(6''-E-p-coumaroylglucoside) | 17.64680825 | 18.06348971 | 0.018727399 | 0.749145857 |
| O-Ethyl O-(4-nitrophenyl) phenylphosphonothioate | 21.60014889 | 21.92939872 | 0.025171767 | 0.795950252 |
| 9-Octadecenamide, N-(2-hydroxyethyl)-, (9Z)- | 19.500083 | 19.20433146 | 0.011252046 | 1.227524259 |
| Docosahexaenoic acid | 25.51685633 | 25.18658212 | 0.046719527 | 1.25725231 |
| 3,6-Epoxy-5,5',6,6'-tetrahydro-b,b-carotene-3',5,5',6'-tetrol | 18.49290987 | 18.91887464 | 0.046366205 | 0.744340802 |
| Eicosapentaenoic Acid | 21.57088341 | 21.13623648 | 0.04402177 | 1.351580021 |
| Myotoxin A | 22.42873879 | 22.21890904 | 0.000120177 | 1.156551692 |
| PC(14:0/2:0) | 18.77991556 | 19.10897636 | 0.021202177 | 0.796054548 |
| 1alpha,25-dihydroxy-26,26, 26,27,27,27-hexamethylvitamin D3 | 22.88669159 | 23.23978601 | 0.039846473 | 0.782903052 |
| Lumichrome | 17.10130364 | 17.47885605 | 0.033179859 | 0.769742382 |
| Myristic acid | 19.03317817 | 18.70543827 | 0.030928158 | 1.255045701 |
| N-Nervonoyl Tryptophan | 23.02171926 | 23.35801192 | 0.024921799 | 0.792074115 |
| PC(O-18:2/2:0) | 21.55792304 | 21.91668197 | 0.014685939 | 0.779835138 |
| DL-13-hydroxy stearic acid | 22.37530327 | 22.05979017 | 0.027661624 | 1.24445417 |
| D-erythro-L-galacto-Nonulose | 24.79432887 | 25.10774145 | 0.033352302 | 0.804735968 |
| Phellogenic acid | 20.77921471 | 21.15776988 | 0.042616311 | 0.769207547 |
| 4Z-Nonenal | 19.68329988 | 20.00095423 | 0.01908669 | 0.802373378 |
| Trichothec-9-ene-4.beta.,15-diol, 12,13-epoxy- | 18.51703945 | 18.79515652 | 0.004775074 | 0.824666628 |
| Isopropyl N-acetoxy-N-phenylcarbamate | 21.69397011 | 21.90683399 | 8.6434E-05 | 0.862822748 |
| 1-(1,2,3,4,5-Pentahydroxypent-1-yl)-1,2,3,4-tetrahydro-beta-carboline-3-carboxylate | 18.31955112 | 18.03741701 | 0.00579951 | 1.21599231 |
| (E)-N'-(3-allyl-2-hydroxybenzylidene)-2-(4-benzylpiperazin-1-yl)acetohydrazide | 18.21689801 | 18.56089542 | 0.041683273 | 0.787855301 |
| Tetraethylene glycol monododecyl ether | 20.43912466 | 20.71876858 | 0.02034644 | 0.82379432 |
| Leu-Ala-Ile | 18.24811368 | 18.58179031 | 0.032809694 | 0.793511683 |
| p-Mentha-1,3,8-triene | 17.37062775 | 17.67703604 | 0.046820328 | 0.808652466 |
| Niludipine | 20.9713991 | 20.75331921 | 0.000270841 | 1.163184447 |
| 8-[1]-ladderane octanoic acid | 20.36267136 | 20.03306601 | 0.032004053 | 1.256669562 |
| Uridine | 20.8615433 | 20.58080894 | 0.006913686 | 1.214813095 |
| L-Sorbose | 27.87250755 | 27.54008156 | 0.012979498 | 1.259128913 |
| Pteroside A | 17.70463666 | 18.0086724 | 0.022803389 | 0.809983411 |
| Tris(2-ethylhexyl) phosphate | 20.75067026 | 21.02567665 | 0.01175367 | 0.826446659 |
| N,N-Diethylglycine | 28.63576934 | 28.92080848 | 0.025409732 | 0.820719344 |
| 5Z,13Z,16Z,19Z-Docosatetraenoic acid | 20.84474799 | 20.55734544 | 0.016554379 | 1.220440998 |
| Ethylphenidate | 18.75217003 | 18.5004238 | 0.002179655 | 1.190647394 |
| 2,5-Dihydro-1H-pyrrole-2-carboxylic acid | 24.45870666 | 24.68768085 | 0.000849002 | 0.853241367 |
| N-[9,10-Dihydrojasmonoyl]isoleucine | 17.11325983 | 17.39699224 | 0.019115395 | 0.821463051 |
